# Catalytic reduction of 4-nitrophenol using 2D-molybdenum ditelluride

**DOI:** 10.1039/d5dt01874a

**Published:** 2025-10-13

**Authors:** Arun Kumar Samuel, Zeliha Ertekin, B. Moses Abraham, Zhengxin Yang, Mark D. Symes, Alexey Y. Ganin

**Affiliations:** a WestCHEM, School of Chemistry, University of Glasgow, University Avenue Glasgow G12 8QQ UK; b Departament de Ciència de Materials i Química Física & Institut de Química Teòrica i Computacional (IQTCUB), Universitat de Barcelona c/Martí i Franquès 1-11 08028 Barcelona Spain alexey.ganin@glasgow.ac.uk

## Abstract

The catalytic reduction of 4-nitrophenol to 4-aminophenol is a crucial reaction in the synthesis of aromatic amines, which serve as key intermediates in the chemical and pharmaceutical industries. This study explores the phase engineering and catalytic performance of molybdenum ditelluride (MoTe_2_) synthesized *via* a solid-state reaction. By optimizing annealing conditions, a mixed 1T′/2H-MoTe_2_ phase structure was successfully achieved, exhibiting significantly enhanced catalytic efficiency compared to the individual 1T′-monoclinic and 2H-hexagonal phases. The mixed-phase MoTe_2_ catalyst facilitated over 80% reduction of 4-nitrophenol within 15 minutes, as confirmed by UV-Vis spectroscopy. Further validation through ^1^H-NMR and LC-MS provided comprehensive insights into product identification, yield quantification, and potential by-product formation. Experimental results, supported by density functional theory (DFT) calculations, demonstrated that the mixed 1T′/2H phase of MoTe_2_ possesses favorable thermodynamic properties, particularly in hydrogen adsorption. These findings highlight the potential of phase-engineered MoTe_2_ as an efficient catalyst for amine synthesis application.

## Abstract 2

MoTe_2_ facilitates the catalytic reduction of 4-nitrophenol to 4-aminophenol. This transformation is important in the synthesis of aromatic amines, which function as essential intermediates in both chemical and pharmaceutical manufacturing.

## Graphical abstract

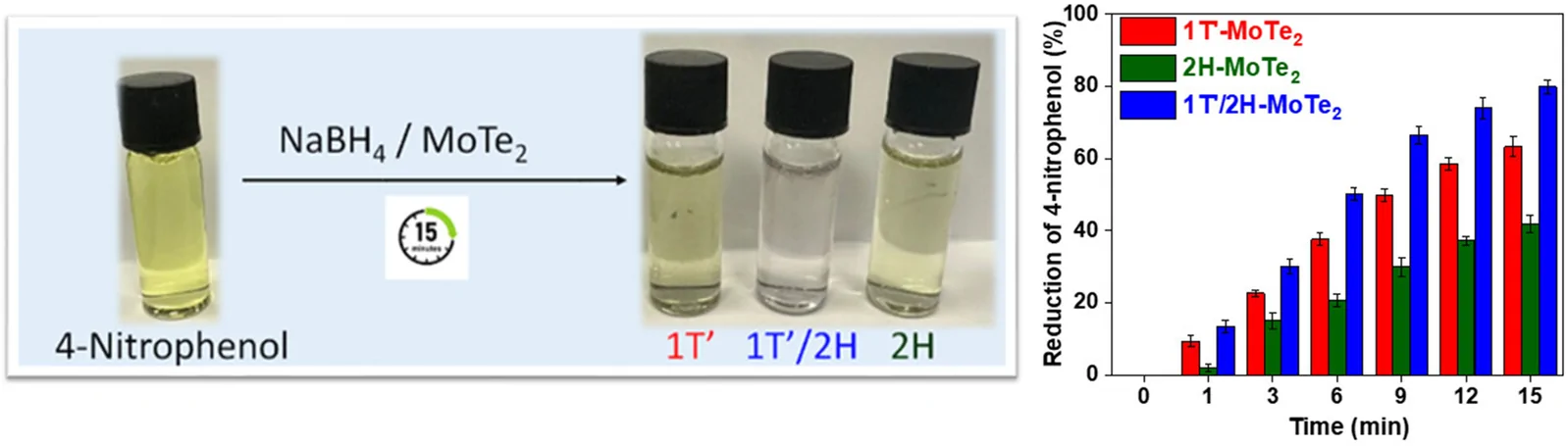

## 1. Introduction

The conversion of 4-nitrophenol into 4-aminophenol is essential, as 4-aminophenol is a key intermediate in the synthesis of pharmaceuticals, dyes, and other chemicals.^1^ This process, crucial for producing aromatic amines, is primarily achieved through catalytic hydrogenation and chemical reduction processes.^1,2^ Catalytic reduction is often favored over catalytic hydrogenation for nitrophenol reduction due to its operational simplicity, safety, and cost efficiency. Unlike catalytic hydrogenation, which requires high pressure and the handling of flammable hydrogen gas, catalytic reduction uses milder conditions and safer reducing agents such as sodium borohydride (NaBH_4_). Furthermore, catalytic reduction generally offers higher selectivity and yields, producing fewer by-products and enhancing the overall reaction efficiency. It also exhibits better compatibility with various functional groups, making it more versatile for complex synthetic applications.^3,4^ Metal nanoparticles, particularly noble metals such as gold (Au), palladium (Pd), silver (Ag), and platinum (Pt), have emerged as effective catalysts for the conversion of 4-nitrophenol to 4-aminophenol.^3,5^ Their high specific surface area promotes rapid reaction rates and selectivity, thereby minimizing by-product formation. However, these nanoparticles are susceptible to aggregation due to their nanoscale dimensions and elevated surface energy, which can compromise their catalytic efficiency over time.^2^ Moreover, complexities like separating and recycling noble metal nanoparticles, particularly those modified with surfactants to mitigate aggregation, pose significant challenges.^6^ Strategies such as anchoring nanoparticles onto supports like silica^7^ or carbon^8^ can enhance stability but complicate the catalyst recovery process.

Two-dimensional (2D) metal chalcogenides, such as molybdenum disulfide (MoS_2_), have been extensively studied as catalysts for the reduction of 4-nitrophenol, primarily due to their high surface area, dense distribution of active sites, and good electron mobility.^9,10^ However, MoS_2_ in its pure form is limited by its intrinsic electronic structure, which restricts its catalytic efficiency and necessitates modifications to optimize its performance. To enhance MoS_2_ catalytic efficiency, various strategies have been employed, including doping with metals^11–13^ and/or blending it with metal oxides.^14,15^ For instance, Ni-doped MoS_2_ has demonstrated improved catalytic activity due to the increased interlayer spacing and the resulting structural and electronic modifications.^16^ Similarly, MoS_2_-based nanocomposites, such as MoS_2_/ZnO, have shown enhanced electron transfer, accelerating the reduction of 4-nitrophenol.^17^ Additionally, composites like CdS-MoS_2_/rGO (where rGO is reduced graphene oxide) have exhibited notable efficiency in photocatalytic reduction, where the synergy of MoS_2_ with reduced graphene oxide facilitates effective electron–hole pair separation.^18^ While these modifications have led to significant improvements in MoS_2_ catalytic performance, the catalytic potential remains constrained by the need for such external modifications. This limitation has driven the search for alternative catalysts with superior intrinsic properties.

Molybdenum ditelluride (MoTe_2_), a structural analog of MoS_2_, offers several key advantages in catalytic applications. MoTe_2_ exhibits a higher density of electroactive sites, enhancing^19^ catalytic activity by providing more surface area for reactant interaction.^20^ It also possesses superior electrical conductivity compared to MoS_2_, facilitating more efficient electron transfer, especially in reactions requiring rapid electron movement.^21^ Additionally, MoTe_2_ polymorphism provides an opportunity for phase engineering (1T′-monoclinic and 2H-hexagonal),^22,23^ where different crystalline phases with distinct structural and electronic properties can be tailored to improve catalytic performance.^24,25^ By optimizing these phases, MoTe_2_ electroactive sites, conductivity, and stability can be precisely controlled, making it highly adaptable for specific catalytic reactions. This flexibility in phase and structural tuning gives MoTe_2_ a clear advantage over MoS_2_, suggesting superior catalytic performance could be achieved, particularly in 4-nitrophenol reduction, while minimizing the need for additional modifications.

Therefore, this study provides the first comprehensive comparison and in-depth investigation of MoTe_2_ polymorphs as catalysts for 4-nitrophenol reduction, highlighting their catalytic efficacy and underlying reaction mechanisms. 1T′-MoTe_2_ and 2H-MoTe_2_ polymorphs (as well as a mixed 1T′/2H phase) were synthesized using a solid-state reaction method, with controlled annealing enabling the formation of distinct phases.^19^ Physicochemical characterizations including XRD, Raman, SEM, and XPS techniques were employed to provide insights into the structural and morphological properties of the MoTe_2_ phases. Unlike traditional UV-Vis spectroscopy (methods used previously for product verification), this research employed ^1^H Nuclear Magnetic Resonance (^1^H NMR) and Liquid Chromatography-Mass Spectrometry (LC-MS) to accurately quantify the conversion of 4-nitrophenol and confirm the reaction products. This rigorous analytical approach validated the reduction of 4-nitrophenol to 4-aminophenol and enabled precise yield quantification. Among the MoTe_2_ samples tested, the mixed-phase catalyst showed the best catalytic performance, achieving over 80% reduction with a molar ratio of MoTe_2_ to 4-nitrophenol of ∼2.85. This performance was achieved without the need for high surface area carbon-based composites or noble metal composites. Detailed density functional theory (DFT) calculations further elucidated the favorable thermodynamics for the reaction exhibited by the mixed 2H/1T′ phases, particularly their enhanced hydrogen surface adsorption, suggesting a rationale for their catalytic potential.

## 2. Experimental

### 2.1. Preparation of MoTe_2_

1T′-MoTe_2_ powder was synthesized by placing 2 mmol of Mo (99.95%, Sigma Aldrich) and 4 mmol of Te lumps (99.99%, Alfa Aesar) into a 9 mm Pyrex ampoule, which was then evacuated to a pressure of 4.2 × 10^−2^ mbar and flame-sealed. To ensure a uniform mixture, the ampoule was gently shaken. The ampoule was heated to 400 °C at a rate of 1 °C per minute and temperature was held for 16 hours in a muffle furnace, followed by natural cooling to room temperature. The product was ground and re-annealed using the same heating (400 °C for 16 hours) protocol. The mixed 1T′/2H-MoTe_2_ and 2H-MoTe_2_ powders were synthesized using the same experimental procedure, except the reaction temperatures used were 450 °C and 550 °C, respectively.

### 2.2. Characterization of MoTe_2_ polymorphs

Powder X-Ray Diffraction (PXRD) analysis was conducted using the Rigaku Miniflex X-ray diffractometer with advanced detector (600 W X-ray tube, D/teX Ultra silicon strip detector). Raman spectroscopy (Horiba Jobin-Yvon LabRam HR800) measurements were carried out with a 532 nm laser. To prevent sample degradation, an aperture size of 100 μm and a 1% filter were applied. Scanning Electron Microscopy (SEM) and energy-dispersive X-ray analysis (EDAX) images were captured using a TESCAN CLARA microscope, coupled with an Oxford Instruments UltimMax 65 detector featuring the Aztec live interface. X-ray photoelectron spectroscopy (XPS) analysis was performed on a Shimadzu (AXIS Supra+) instrument, coupled with Al K_α1_ radiation.

### 2.3. The catalytic reduction of 4 nitro-phenol

Reduction of 4-nitrophenol employing sodium borohydride (NaBH_4_) as the reducing agent was carried out using MoTe_2_ catalysts, namely 1T′-MoTe_2_, 2H-MoTe_2_ and a mixed 1T′/2H MoTe_2_. A solution containing 10 mL of 4-nitrophenol (0.5 mM, Merck, >99%) and NaBH_4_ (0.13 mM, Sigma–Aldrich, 98%) was prepared, and one of the following catalysts was added individually: 1T′-MoTe_2_, 1T′/2H-MoTe_2_, or 2H-MoTe_2_ (1.42 μM each). The solution was stirred at room temperature (∼20 °C). The catalyst-to-reactant molar loading was then calculated for each MoTe_2_ catalyst, with a molar ratio (mol/mol) of MoTe_2_ to 4-nitrophenol of approximately 2.85. The reduction progress of 4-nitrophenol was monitored using a UV–Vis spectrophotometer (Agilent Cary 60) in the wavelength range of 200–500 nm. Typically, 200 μL of the reaction solution was sampled at specific time intervals (1, 3, 6, 9, 12, and 15 minutes) and diluted to 3 mL before measurements. More details on catalytic reduction of 4 nitro-phenol are given in SI section 1.

The reduction of 4-nitrophenol to 4-aminophenol was also analyzed using ^1^H nuclear magnetic resonance (^1^H NMR) spectroscopy (400 MHz, Bruker). The water suppression method was used to record the ^1^H NMR spectra, with 500 μL of sample solution mixed with 100 μL of dimethyl sulfoxide-d_6_ (DMSO-d_6_, [67-68-5], 99.9% D, Cambridge Isotope Laboratories, Inc.). To quantify the removal of 4-nitrophenol, the liquid products obtained from the reduction reaction were analyzed. After filtering, the resulting liquid was analyzed using an Agilent high-performance liquid chromatography (HPLC) system equipped with a Phenomenex C18 column (15 cm length). The mobile phase consisted of 0.1% formic acid in water (Fisher chemical, Optima™ LC/MS Grade) and methanol (Fisher chemical, Optima™ LC/MS Grade) in an 80 : 20 ratio. The HPLC system operated at a flow rate of 1 mL min^−1^, maintained at 60 °C, with a detector wavelength of 278 nm. To further identify the reaction products, mass spectra of the detected intermediates were obtained using a high-resolution Agilent 6545 QTOF mass spectrometer coupled to an Agilent Infinity 1290 UHPLC system. For the reusability test of the catalyst (1.42 μM), it was reused without filtration and centrifuged at 4000 rpm for 5 minutes. The catalyst was then carefully separated, the supernatant was removed, the solid was washed, centrifuged again, and the supernatant was removed once more. The recovered catalyst was subsequently mixed with 4-nitrophenol (0.5 mM) and NaBH_4_ (0.13 mM) at room temperature (∼20 °C) for 15 minutes. This cycle was repeated five times to evaluate the catalyst's reusability.

## 3. Results and discussion

### 3.1 Physical characterization

Fig. 1a shows the powder X-ray diffraction (PXRD) pattern of MoTe_2_ powder synthesized at 400 °C, revealing the emergence of 1T′-MoTe_2_, consistent with prior studies.^25^Fig. 1b showcases Raman bands at 80.5, 108.2, 128.5, 161.3, and 250.4 cm^−1^, corresponding to the characteristic A_g_ modes of 1T′- MoTe_2_.^23,26^ The PXRD pattern of the sample prepared at 450 °C suggests the formation of a mixed 1T′/2H-MoTe_2_ phase, identifiable by additional peaks corresponding to the 2H-phase (Fig. 1a). The presence of Raman bands at 119.1 cm^−1^ and 232.4 cm^−1^ confirms the existence of the 2H-MoTe_2_ phase, while a Raman peak at 161.3 cm^−1^ (A_g_ mode of 1T′) corroborates the coexistence of the mixed 1T′/2H phases.^23^ This suggests that the annealing temperature at 450 °C is inadequate for achieving sufficient Te vapor pressure, impeding the complete transformation to the 2H-phase.^26^ The PXRD pattern of the sample prepared at 550 °C confirms the formation of 2H-MoTe_2_ (Fig. 1a). The 2H-MoTe_2_ phase is confirmed by distinctive Raman bands at 119.8, 172.6, and 232.6 cm^−1^ (Fig. 1b). Subsequent SEM analysis showed that there is variability in crystallite sizes across three samples with the mixed-phase material showing the smallest sizes (Fig. 1c–e). However, as the crystallite sizes in all three samples are between 5–10 μm, it is unlikely that the morphology differences would have a significant impact on catalytic performance. In particular, mixed 1T′/2H phase showed relatively smaller and porous structure as compared to 1T′ and 2H. Elemental mapping confirms a homogeneous distribution of Mo and Te elements irrespective of the MoTe_2_ polymorphs formed (Fig. S1) and the elemental compositions are summarized in SI Table S1. To further support this conclusion, we have included TEM analysis (Fig. S2). Due to the limited magnification of the TEM (not high-resolution), SAED and fully resolved lattice fringes could not be clearly obtained; nevertheless, the TEM images reveal distinct contrast variations and domain-like features across the samples. Notably, in certain regions of the mixed 1T′/2H sample (Fig. S2e), two different lattice fringe patterns can be discerned, which may correspond to the coexistence of distinct phases. These observations are consistent with the presence of multiple structural phases within MoTe_2_ evidenced from XRD and Raman.

**Fig. 1:**
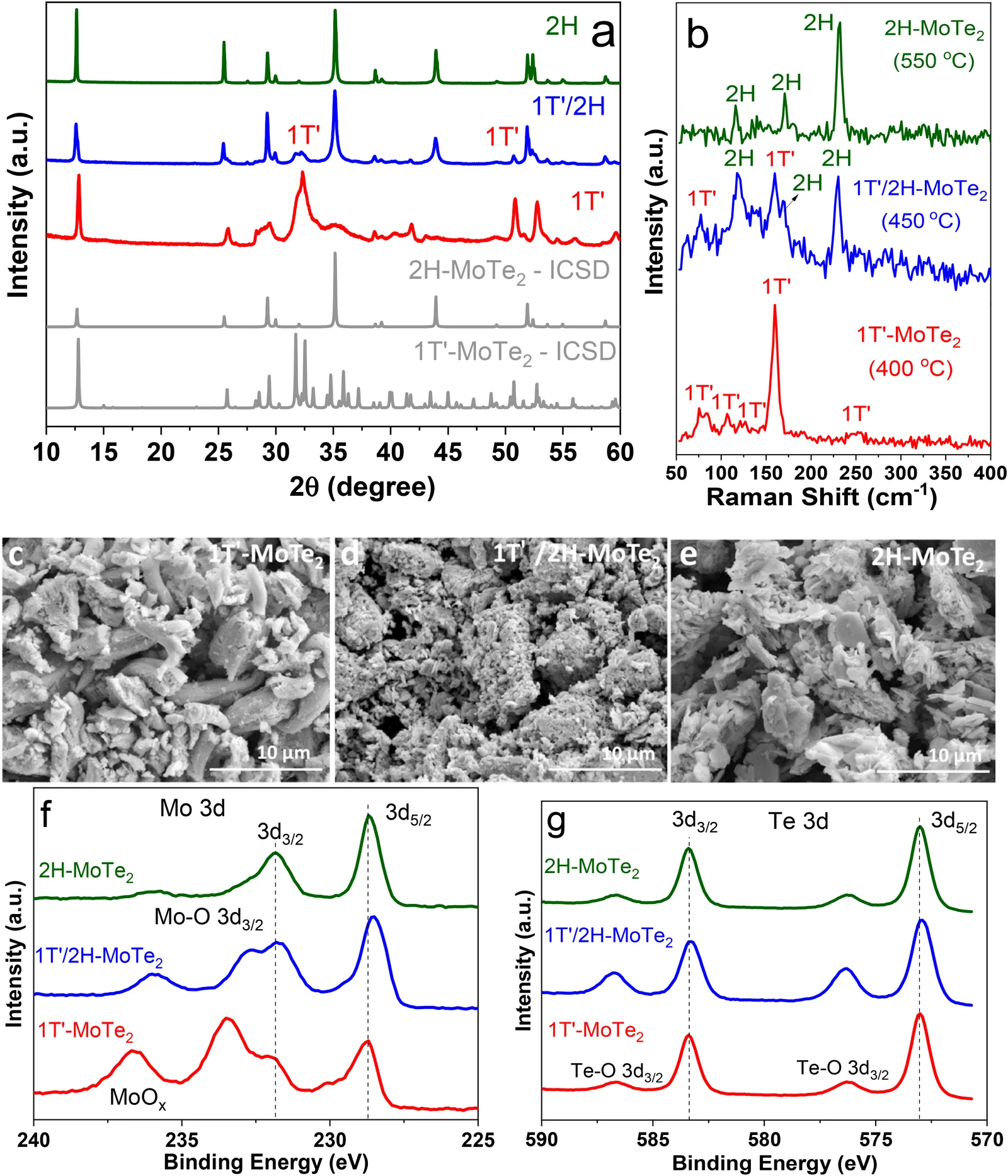
(a) PXRD patterns of MoTe_2_ at different temperatures. (b) Raman spectra confirm the formation of 1T′-MoTe_2_, mixed 1T′/2H-MoTe_2_ and 2H-MoTe_2_ phases. SEM images of (c) 1T′-MoTe_2_, (d) mixed-phase 1T′/2H-MoTe_2_ and (e) 2H-MoTe_2_. XPS spectra of MoTe_2_ showing (f) Mo 3d and (g) Te 3d peaks at different annealing temperatures.

XPS analysis was employed to elucidate the chemical composition, electronic states, and surface oxidation of the MoTe_2_ powders synthesized at the various temperatures. Fig. S3 illustrates the XPS survey spectra for 1T′-MoTe_2_, 1T′/2H MoTe_2_, and 2H-MoTe_2_, while Fig. 1f and g present high-resolution core-level XPS spectra for the Mo 3d and Te 3d levels, respectively. In the case of 1T′-MoTe_2_ annealed at 400 °C, the Mo 3d_5/2_ and Mo 3d_3/2_ peaks at 225.7 eV and 230.4 eV, respectively, were identified, along with a weak peak at 236.6 eV indicating partial oxidation of MoTe_2_ in air (Fig. S4). The Te 3d peaks at 570.2 eV and 580.6 eV correspond to Te 3d_5/2_ and Te 3d_3/2_, respectively, with a weak peak at 586.5 eV attributed to TeO_*x*_ formed by oxidation (Fig. S4), in agreement with previous reports.^19,27^ For the mixed-phase 1T′/2H-MoTe_2_ annealed at 450 °C, there is a slight variation in the peak trend, particularly in the Mo 3d_3/2_ region. The Mo 3d_3/2_ peak is enhanced, and oxidation peaks are suppressed compared to the 1T′-phase (Fig. 1f). In the case of 2H-MoTe_2_ annealed at 550 °C, dominant Mo 3d peaks at 225.4 eV and 230.4 eV are observed, with minimal oxidation compared to other phases (Fig. 1f).

### 3.2 Catalytic activity

The reduction of 4-nitrophenol (0.5 mM) was conducted using MoTe_2_ catalysts (1.42 μM) and NaBH_4_ (0.13 mM) over a 15-minute duration at room temperature, as illustrated in Fig. 2. The molar ratio of catalyst to 4-nitrophenol is approximately 2.85. The calibration curve of 4-nitrophenol absorbance at various concentrations is presented in Fig. S5. Initially, the aqueous solution of 4-nitrophenol exhibited an absorption peak at 317 nm, accompanied by a yellow coloration. The introduction of NaBH_4_ induced a shift in the absorbance peak from 317 nm to 400 nm, indicating the formation of the 4-nitrophenolate ion within an alkaline environment (pH = 10.2, see Fig. 2). Concurrently, the solution changed from a pale to a vibrant yellow. In the absence of a catalyst, the absorbance peak at 400 nm remained constant, confirming that NaBH_4_ alone did not cause the reduction process (Fig. S6). However, with the addition of the 1T′/2H-MoTe_2_ catalyst (Fig. 2b), the peak at 400 nm gradually diminished. Simultaneously, a new peak with absorption maxima at 298 nm and 231 nm emerged, indicating the presence of 4-aminophenol. After 15 minutes, the intensity of the peak at 400 nm had diminished significantly, while the peak at 298 nm had intensified, signifying the reduction of 4-nitrophenol to 4-aminophenol.

**Fig. 2:**
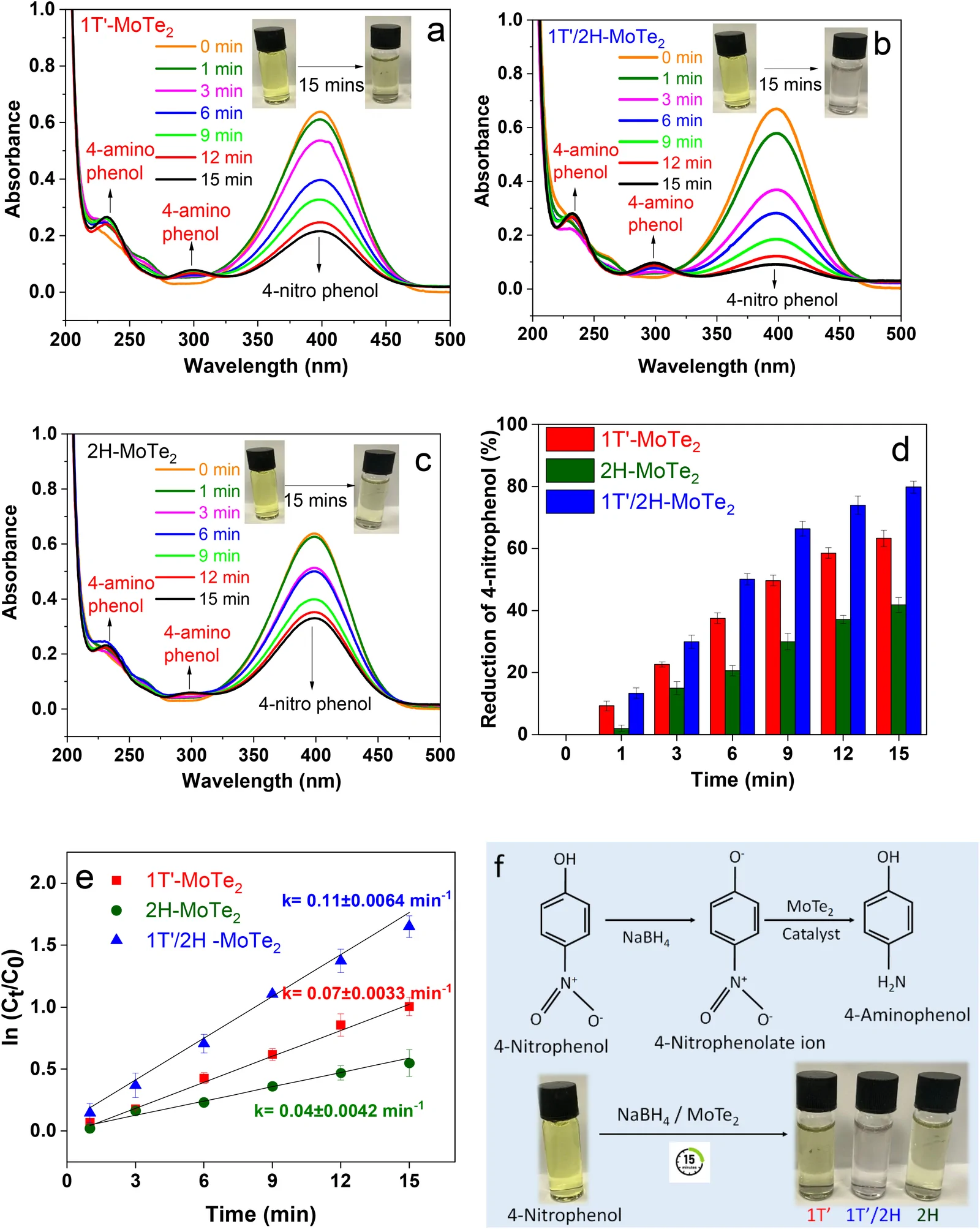
Time-dependent UV–visible absorption spectra of 4-nitrophenol (0.5 mM) reduced by NaBH_4_ (0.13 mM) in the presence of (a) 1T′-MoTe_2_, (b) mixed 1T′/2H-MoTe_2_ and (c) 2H-MoTe_2_ catalysts. (d) The effect of reaction time on the consumption of 4-nitrophenol and (e) Langmuir–Hinshelwood plot of ln(*C*_*t*_/*C*_0_) over time using 1T′-MoTe_2_, 2H-MoTe_2_, and 1T′/2H-MoTe_2_ catalysts. (f) Pictograph showing the 4-nitrophenol reduction using MoTe_2_ polymorphs.

Fig. 2d illustrates the time-dependent study results for all the MoTe_2_ polymorphs under controlled conditions. In the absence of a catalyst (Fig. S6), no reduction of 4-nitrophenol occurred. However, the presence of the MoTe_2_ catalyst significantly facilitated the reduction process. A total reaction time of 15 minutes was chosen for direct comparison with analogous reactions reported with other 2D-TMDs. Although there is no data available for 2D-MoTe_2_ compounds in the reduction of 4-nitrophenol, similar family compounds, such as MoS_2_, have been tested for reduction efficiency. These studies reported a ∼55% extent of reduction in 15 minutes using a catalyst amount of 1000 mg L^−1^, which is double the amount used in this study.^17^ The 1T′-MoTe_2_ catalyst exhibited a 63% reduction in 4-nitrophenol (Fig. 2a), while the 2H-MoTe_2_ catalyst demonstrated a reduction percentage of 43% (Fig. 2c). The mixed 1T′/2H-MoTe_2_ catalyst displayed the fastest reduction, surpassing 80% conversion of 4-nitrophenol within 15 minutes (Fig. 2b). Based on the percentage reduction of 4-nitrophenol, the catalytic activity of 2D MoTe_2_ follows the order: mixed-phase 1T′/2H-MoTe_2_ > pure 1T′-MoTe_2_ > 2H-MoTe_2_. This suggests that the presence of mixed oxidation states in both the pure 1T′-MoTe_2_ and the mixed 1T′/2H-MoTe_2_ phases plays a significant role in enhancing catalytic performance, as supported by the XPS analysis (Fig. 1f). Consistent with this observation, Sun *et al.* reported that mixed-valence states improve catalytic activity by facilitating more effective electron transfer from the reducing agent (NaBH_4_) to 4-nitrophenol, thereby accelerating the reduction reaction.^28^

To further understand the reduction of 4-aminophenol using this mixed catalyst, ^1^H NMR and LC-MS analyses were carried out. The ^1^H NMR spectra of 4-nitrophenol and 4-aminophenol in DMSO-d_6_ are provided in the SI as Fig. S7 and S8, respectively. The addition of NaBH_4_ caused a shift in the ^1^H NMR peaks, indicating the formation of the 4-nitrophenolate ion (Fig. 3). This formation was also confirmed by a corresponding shift in the absorbance peak observed in UV measurements. It should be noted that the initial concentration of 4-nitrophenol (0.5 mM) was too low for effective ^1^H NMR analysis. Therefore, to facilitate the analysis of the obtained product, the concentration of 4-nitrophenol was increased tenfold, allowing for the successful detection of 4-aminophenol. Under these conditions, the high concentration of NaBH_4_ and hydrogen generation from NaBH_4_ in the presence of a catalyst may affect the ^1^H NMR chemical shifts of 4-aminophenol (Fig. 3d). Fig. 3a presents the ^1^H NMR spectrum of a mixture containing commercial 4-nitrophenol and 4-aminophenol in the presence of NaBH_4_, with the purpose of comparing the spectra before and after the reduction process to confirm the reaction's progress and product formation. The peaks in the ^1^H NMR spectrum are consistent with literature values, showing two doublets at 6.54 and 6.47 ppm, which correspond to the two pairs of aromatic protons in the 4-aminophenol molecule. Additionally, two doublets at 6.32 and 7.89 ppm are attributed to the aromatic protons of 4-nitrophenol.^29^ The ^1^H NMR spectrum of commercial 4-aminophenol in the presence of NaBH_4_ is also shown in Fig. 3b. Fig. 3c displays the ^1^H NMR spectrum of the solution before adding the catalyst, confirming the structure of 4-nitrophenol. Fig. 3d shows the ^1^H NMR spectrum of the 4-aminophenol after 15 minutes in the presence of the mixed 1T′/2H-MoTe_2_ catalyst, and confirms that no peaks corresponding to 4-nitrophenol are present after the reaction, showing it was fully converted.

**Fig. 3:**
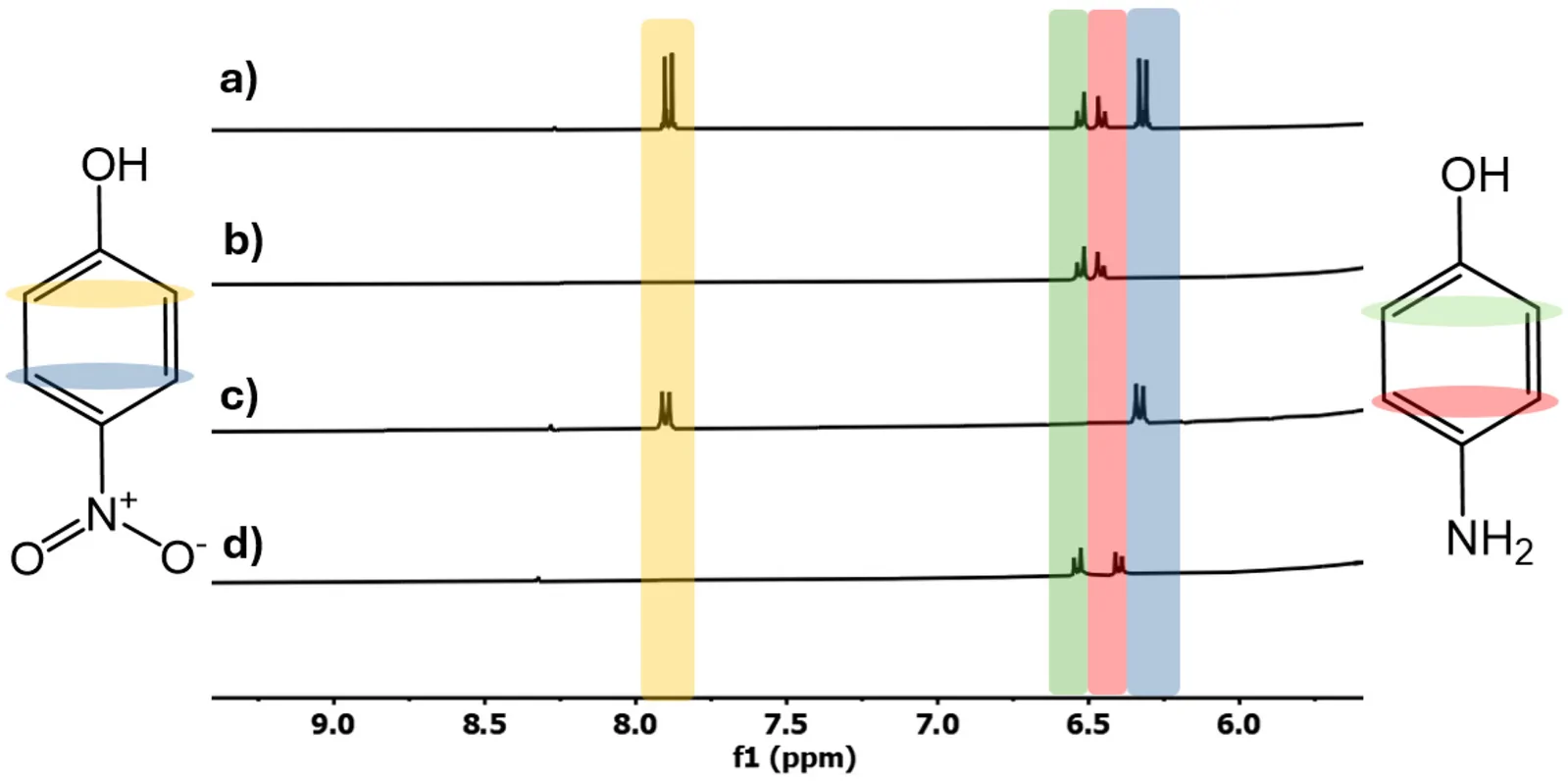
^1^H NMR spectra of: (a) commercial 4-nitrophenol (5.0 mM) and commercial 4-aminophenol (5.0 mM) in the presence of NaBH_4_, before catalyst addition (1.3 M); (b) commercial 4-aminophenol (5.0 mM) in the presence of NaBH_4_ (1.3 M); (c) commercial 4-nitrophenol (5.0 mM) in the presence of NaBH_4_ (1.3 M) before catalyst addition; and (d) after 15 minutes of reduction using mixed-phase 1T′/2H-MoTe_2_ catalyst (14 mM).

To further confirm and better quantify the conversion of 4-nitrophenol, the liquid products from the reduction reaction were also analyzed using LC-MS (Fig. S9). The reaction was carried out with the 1T′/2H-MoTe_2_ catalyst (1.42 μM) and NaBH_4_ (0.13 mM) at room temperature for 15 minutes. After the reaction, the mixture was filtered, and the resulting liquid was then analyzed. The concentration of 4-nitrophenol in the sample was determined by comparing the peak area to a calibration curve (Fig. S10a). This curve was constructed using chromatograms of standard 4-nitrophenol solutions. The corresponding mass spectrum for the characteristic peak (retention time = 2.64 min) in the chromatogram is also provided in Fig. S10b. The analytical calibration involved using five standard solutions with concentrations of 0.5, 1.0, 3.0, 4.0, and 5.0 mM (Fig. S9a). By comparing the peak areas of 4-nitrophenol before and after the reaction using the calibration curve, it was calculated that the 1T′/2H-MoTe_2_ catalyst removed 88% of the 4-nitrophenol, which matches the UV measurements closely (Fig. 2b).

The reusability of a catalyst is a critical factor for its practical application. Therefore, the degradation of 4-nitrophenol using 1T′/2H-MoTe_2_, which demonstrated the best catalytic activity, was evaluated over five cycles (Fig. S11).

### 3.3 Mechanistic studies

The reaction mechanism for the reduction of 4-nitrophenol to 4-aminophenol catalyzed by MoTe_2_ is shown in SI Fig. S12. The reduction of 4-nitrophenol by borohydride to 4-aminophenol involves a six-electron transfer process, with electrons moving from BH_4_^−^ to 4-nitrophenol through the adsorption of reactant molecules on the catalyst's surface.^30^ The positively charged catalyst plays a crucial role by facilitating the hydrolysis of BH_4_^−^ to generate active hydrogen species and by catalyzing the hydrogenation of 4-nitrophenol to 4-aminophenol.^31^ Although NaBH_4_ can release H_2_ through hydrolysis (NaBH_4_ + 2H_2_O → NaBO_2_ + 2H_2_), the hydrogen formed *in situ* can remain associated with the catalyst surface as surface-active hydrogen, which participates in the hydrogenation step.^31^ Sodium borohydride chemisorbs on the catalyst surface, transferring electrons or hydride species directly to 4-nitrophenol. Protons from the aqueous medium combine with these electrons or surface hydrogen to complete the reduction, yielding 4-aminophenol.^32,33^ Thus, the catalyst mediates both electron transfer from NaBH_4_ and the activation of hydrogen species on its surface. A similar mechanism has been reported for 4-nitrophenol reduction over copper hexacyanoferrate nanocrystals, supporting the proposed pathway.^31^ Moreover, the energy band bending at the junction between 1T′ and 2H phase interface aids in charge transport, thereby expediting the reduction process.^34^ It is also reported that the 1T′/2H-MoTe_2_ plays a crucial role in enhancing the electron relay process due to its high electron mobility.^27,35^ In addition, the structural features of 1T′/2H-MoTe_2_ such as exposed edges and a high density of defects further boost its catalytic activity, enabling it to catalyze the hydrogen evolution reaction (HER) with noble-metal-like performance.^27^

A plausible reaction mechanism for the reduction of 4-nitrophenol on 1T′/2H-MoTe_2_ is proposed based on the Langmuir–Hinshelwood (L–H) model, where both 4-nitrophenol and BH_4_^−^ adsorb onto the catalyst surface. BH_4_^−^ may donate electrons, generating hydrogen species *via* water reduction. These hydrogen species are suggested to reduce adsorbed 4-nitrophenol to 4-aminophenol, which then desorbs to free active sites. While direct evidence of intermediates is lacking and alternative pathways cannot be ruled out, ^1^H NMR (Fig. 3d) and mass spectrometry (Fig. S13) confirm 4-aminophenol as the primary product. Despite the speculative nature of this plausible mechanism, the catalyst shows high activity without noble metal additives.^36–38^

Computational studies analyzing hydrogen adsorption on the surface of 1T′-MoTe_2_ have been previously reported in the literature.^25^ Building on this, the present work focuses on studying the reduction process facilitated by the adsorption of hydrogen on the catalyst surface. The investigation involves comparing the presence of H_2_ in three scenarios: (i) 4-nitrophenol + 1T′/2H-MoTe_2_, (ii) NaBH_4_ + 1T′/2H-MoTe_2_, and (iii) 4-nitrophenol + NaBH_4_ + 1T′/2H-MoTe_2_ (Fig. 4a), with meticulous GC measurement for H_2_ calibration (Fig. S14). As anticipated, the combination of 4-nitrophenol with 1T′/2H-MoTe_2_, without NaBH_4_, results in minimal H_2_ production. The above results suggest that NaBH_4_ generates hydrogen, which is subsequently adsorbed onto the catalyst's surface and facilitates the hydrogenation reaction. In the presence of 4-nitrophenol + NaBH_4_ + 1T′/2H-MoTe_2_, the hydrogen production is approximately doubled (11.7 nM of H_2_) compared to NaBH_4_ + 1T′/2H-MoTe_2_ (5.5 nM of H_2_), as depicted in Fig. 4b. The addition of NaBH_4_ to 4-nitrophenol results in the formation of the 4-nitrophenolate ion, accompanied by an increase in solution pH from around 5.8 to approximately 10.2 due to the basicity of BH_4_^−^. This reduction reaction is thermodynamically favorable, considering the standard reduction potentials of 4-nitrophenol to 4-aminophenol (*E*° ≈ −0.76 V) and BH_4_^−^ to borates (*E*° ≈ −1.33 V). However, the reaction kinetics are limited by electrostatic repulsion between the negatively charged BH_4_^−^ and the 4-nitrophenolate ion. The catalyst plays a crucial role by facilitating the hydrolysis of BH_4_^−^ to generate active hydrogen species and catalyzing the hydrogenation of 4-nitrophenol to 4-aminophenol *via* surface-adsorbed hydrogen.^31^ Following the 4-nitrophenol reduction experiment, the post-experimental XRD and Raman analyses reveal that the 1T′/2H-MoTe_2_ catalyst demonstrates good stability, with no observable structural degradation (Fig. S15).

**Fig. 4:**
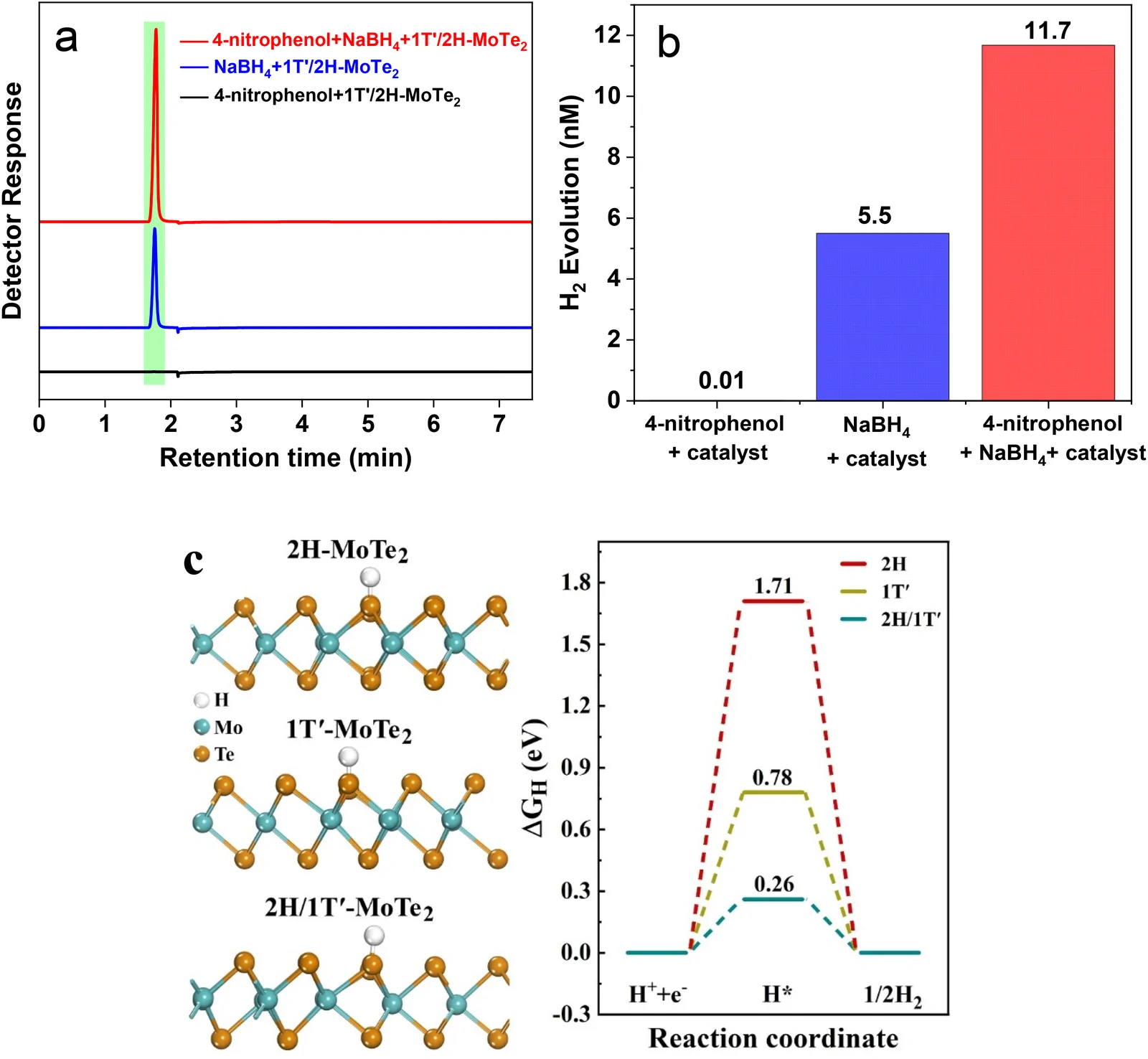
(a) Headspace gas chromatography measurements and (b) hydrogen evolution after 15 minutes of reaction for 4-nitrophenol + 1T′/2H-MoTe_2_ catalyst, NaBH_4_ + 1T′/2H-MoTe_2_ and 4-nitrophenol + NaBH_4_ + 1T′/2H-MoTe_2_ catalyst. (c) The optimized structural configuration of H adsorbed on MoTe_2_ monolayers with 2H, 1T′, and 2H/1T′ phases and the associated free energy diagram.

DFT calculations were conducted to gain deeper insights into the catalytic activity of the studied MoTe_2_ phases. The computational details and a comprehensive description of the DFT studies are provided in SI section 2. Fig. S16 illustrates the optimized structures and corresponding partial density of states of MoTe_2_ monolayers with 2H, 1T′, and 2H/1T′ phases. The band gap of 2H-MoTe_2_ determined using the HSE06 hybrid functional is found to be 1.14 eV. In the case of 1T′-MoTe_2_, the conduction band and valence band states are not completely separated, indicating semimetal characteristics. While, in the 2H/1T′ phase, both the conduction band and the valence band are distinctly separated with a band gap of 0.21 eV, aligning with the experimental results. Typically, the hydrogen atom adsorption on its surface allows for evaluating the performance of a catalyst by examining the associated Gibbs free energy (Δ*G*_H_).^39,40^ An effective catalyst exhibits an intermediate Δ*G*_H_, since the strongly adsorbed hydrogen with a highly negative Δ*G*_H_ can saturate the catalyst surface, while highly positive Δ*G*_H_ presents challenges for hydrogen adsorption onto the catalyst surface by impeding reactivity. Consequently, an ideal hydrogen evolution reaction (HER) catalyst should possess a Δ*G*_H_ value close to 0 eV.^25,41,42^ The relaxed configurations of H adsorbed on MoTe_2_ phases and the associated free energy profile for the hydrogen evolution reaction is shown in Fig. 4c. Clearly, the 2H phase is inactive due to a thermodynamically large uphill step (1.71 eV) for hydrogen adsorption. Although the hydrogen adsorption free energy (Δ*G*_H_) of the 1T′ phase is reduced to 0.78 eV, it still limits H_2_ production activity. In contrast, at the 2H/1T′ interface a Δ*G*_H_ value of 0.26 eV is predicted, representing a significant improvement over both the 2H and 1T′ phases. This demonstrates the potential of manipulating the hydrogen adsorption-free energy by incorporating mixed 2H/1T′ phases to enhance catalytic activity effectively.

Moreover, from EDX analysis (SI Fig. S1) shows noticeable variations in the Te/Mo ratio among 1T′, mixed 1T′/2H, and 2H-MoTe_2_, indicating that Te vacancy formation depends on both the crystalline phase and synthesis method. These vacancies play a key role in modulating the electronic structure and charge distribution. In the mixed 1T′/2H phase, Te vacancies enhance surface adsorption and facilitate electron transfer, improving 4-nitrophenol reduction kinetics. Thus, the catalytic performance is influenced not only by the coexistence of 1T′ and 2H phases but also by the density and distribution of Te vacancies.

## 4. Conclusions

This study examined three different types of MoTe_2_ catalysts and the impact of phase assembly on their catalytic properties for the reduction of 4-nitrophenol. Among them, the 1T′/2H-MoTe_2_ phase exhibited the highest catalytic efficiency, surpassing both monoclinic 1T′-MoTe_2_ and hexagonal 2H-MoTe_2_. The superior performance of 1T′/2H-MoTe_2_ was attributed to its enhanced electron transfer capability and improved charge transport, facilitating the efficient reduction process. LC-MS, UV-Vis spectroscopy, and ^1^H NMR spectroscopy confirmed the successful conversion of 4-nitrophenol to 4-aminophenol, with all techniques yielding consistent results. Additionally, the L–H kinetic model provided insights into the adsorption and desorption mechanisms on the catalyst surface. DFT calculations further supported these findings, revealing that the 2H/1T′ interface exhibited a favorable Δ*G*_H_ value (0.26 eV), optimizing surface adsorption and contributing to the overall catalytic performance enhancement.

## Author contributions

Arun Kumar Samuel: conceptualization, methodology, investigation, data curation, writing – original draft. Zeliha Ertekin: conceptualization, methodology, investigation, data curation, writing – original draft. B. Moses Abraham: investigation, DFT, writing – review & editing. Zhengxin Yang: contributed in TEM characterization & analysis. Mark D. Symes: conceptualization, supervision, writing – review & editing. Alexey Y. Ganin: conceptualization, supervision, writing – review & editing.

## Conflicts of interest

The authors declare that they have no known competing financial interests or personal relationships that could have appeared to influence the work reported in this paper.

## Supplementary Material

DT-054-D5DT01874A-s001

## Data Availability

The data underpinning this study have been deposited in the University of Glasgow's Enlighten database under accession code https://dx.doi.org/10.5525/gla.researchdata.2071. Supplementary information (SI): the catalytic reduction procedure of 4 nitro-phenol (SI section 1) and DFT computational details (SI section 2). It also included data from various analytical techniques, including elemental mapping and EDX (Energy Dispersive X-ray Spectroscopy), elemental analysis, X-ray Photoelectron Spectroscopy (XPS), UV-visible absorption spectroscopy, ^1^H NMR spectroscopy, Liquid Chromatography-Mass Spectrometry (LC-MS), X-ray Diffraction (XRD), and Raman spectroscopy. See DOI: https://doi.org/10.1039/d5dt01874a.
