# Supplementary material for: Catalytic reduction of 4-nitrophenol using 2D-molybdenum ditelluride
Source: Dalton Trans. 2025 Oct 13;54(44):16525–34. doi: 10.1039/d5dt01874a (PMC13725689; doi:10.1039/d5dt01874a)
Supplement: Supplementary file 1 [file DT-054-D5DT01874A-s001.pdf]

Supplementary Information (SI) for Dalton Transactions.
This journal is © The Royal Society of Chemistry 2025

Catalytic Reduction of 4-Nitrophenol Using 2D-Molybdenum Ditelluride

Arun Kumar Samuel[a†], Zeliha Ertekin[a†], B. Moses Abraham[b], Zhengxin Yang[a], Mark D. Symes[a], Alexey Y. Ganin[a*]

[a] *WestCHEM, School of Chemistry, University of Glasgow, University Avenue, Glasgow G12 8QQ, United Kingdom*

[b]*Departament de Ciència de Materials i Química Física & Institut de Química Teòrica i Computacional (IQTCUB), Universitat de Barcelona, c/ Martí i Franquès 1-11, 08028 Barcelona, Spain*

Corresponding author: *alexey.ganin@glasgow.ac.uk

Supplementary Information

Supplementary section 1

The catalytic reduction of 4 nitro-phenol

Reduction of 4-nitrophenol employing sodium borohydride ($NaBH_4$) as the reducing agent was carried out using $MoTe_2$ catalysts, namely 1T'-$MoTe_2$, 2H-$MoTe_2$ and a mixed 1T'/2H $MoTe_2$. A solution containing 10 mL of 4-nitrophenol (0.5 mM, Merck, >99%) and $NaBH_4$ (50 mg, Sigma-Aldrich, 98%) with 1T'-$MoTe_2$, 1T'/2H-$MoTe_2$, and 2H-$MoTe_2$ (5 mg each) powders individually introduced into the solution was stirred at room temperature (~25 °C). The reduction progress of 4-nitrophenol was monitored using a UV–Vis spectrophotometer (Agilent Cary 60) in the wavelength range of 200–500 nm. Typically, 200 µL of the reaction solution was sampled at specific time intervals (1, 3, 6, 9, 12, and 15 minutes) and diluted to 3 mL before measurements. The time-dependent conversion yield of 4-nitrophenol to 4-aminophenol was determined using the equation below:

$$Reduction\ of\ 4-nitrophenol\ (\%) = \frac{(C_0 - C_t)}{C_0} \times 100$$

The reaction kinetics can be approximated by employing the pseudo-first-order reaction approach, as given below:

$$\ln (C_t/C_0) = -kt$$

Here, C_0 and C_t represent the concentration of 4-nitrophenol at the initial time and time t, respectively. The rate of the reaction is determined by examining the slope (represented as 'k'

with units in minutes^{-1}) of the graph plotting ln (C_t/C_0) against time (t in minutes). In this context, 'k' represents the rate constant, which reflects how quickly the reaction is occurring.

Supplementary section 2

Computational details

All calculations were carried out using the Vienna Ab initio simulation package [26], employing the projector augmented wave method [27] via density functional theory (DFT) with the Perdew–Burke–Ernzerhof (PBE) form of the generalized gradient approximation (GGA) [28] to handle the exchange–correlation energy. The Kohn-Sham orbitals were expanded using a plane-wave basis set with a cutoff energy of 520 eV, while van der Waals interactions were considered using the DFT-D3 method [29]. To ensure the total energy convergence, a 5 × 5 × 1 k-point grid using the Monkhorst-Pack scheme [30] was utilized for Brillouin-zone sampling. Optimization of the lattice vectors and atomic positions was conducted to ensure forces on each atom below 0.02 eV/Å and a total energy variation of less than 0.01 meV. To accurately predict band gap values, we employed the Heyd–Scuseria–Ernzerhof (HSE) hybrid functional [31]. Using $MoTe_2$ primitive cell, 3 × 3 × 1 sized supercell was constructed for 2H-, 1T'- and 2H/1T' phases. For the simulation of hydrogen adsorption, we considered monolayered $MoTe_2$ crystals with 15 Å vacuum space in the z-direction. On the $MoTe_2$ surface, the adsorption energies of hydrogen species are determined using the equation:

$$\Delta E_H = E_{MoTe2+H} - E_{MoTe2} - 1/2E_{H2(g)}$$

Here, $E_{MoTe2+H}$ denotes the energy of atomic hydrogen adsorbed on $MoTe_2$. While, E_{MoTe2} and $1/2E_{H2(g)}$ signifies the energies of $MoTe_2$ and hydrogen molecules in the gas phase, respectively. Furthermore, to derive the Gibbs free adsorption energy of the adsorbed atomic hydrogen (ΔG_H), entropy (S) and zero-point energies (E_{ZPE}) are considered, leading to the expression: $\Delta G_H = \Delta E_H + \Delta E_{ZPE} - T\Delta S$.

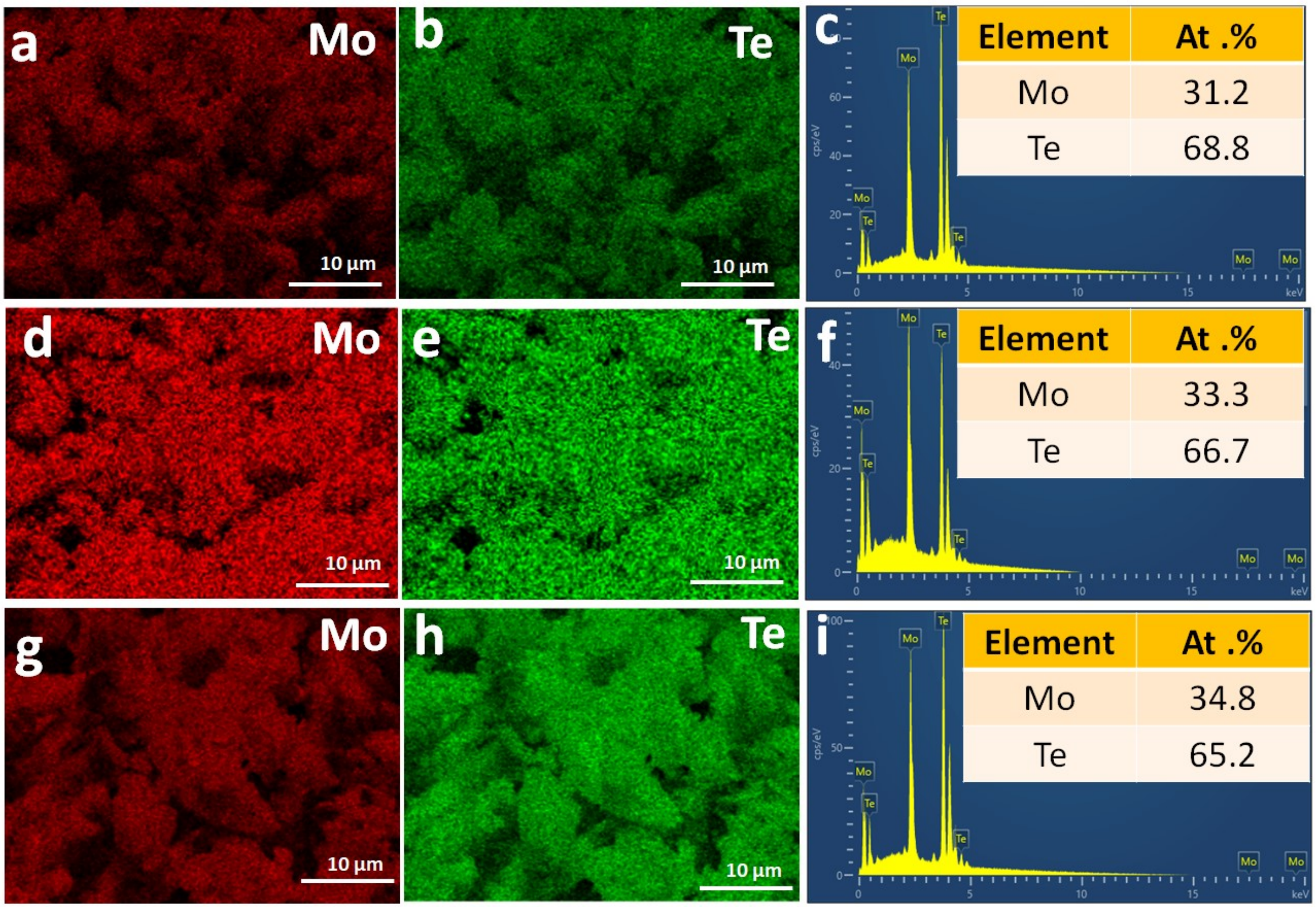

Figure S1. Elemental mapping and EDX spectra of **(a-c)** 1T'-$MoTe_2$, **(d-f)** 1T'/2H $MoTe_2$, and **(g-i)** 2H-$MoTe_2$ powders.

Supplementary Table S1. Elemental analysis of $MoTe_2$ polymorphs was conducted using EDX at various annealing temperatures. Standard deviations were determined by averaging the EDX results from at least five points from each powder samples.

	**Mo, at. %**	**Te, at. %**	**Te / Mo**
Theoretical	33.33	66.67	2.00
1T'-$MoTe_2$	30.22	69.78	2.33 ± 0.34
1T'/2H-$MoTe_2$	34.08	65.92	1.93 ± 0.04
2H-$MoTe_2$	35.16	64.84	1.84 ± 0.05

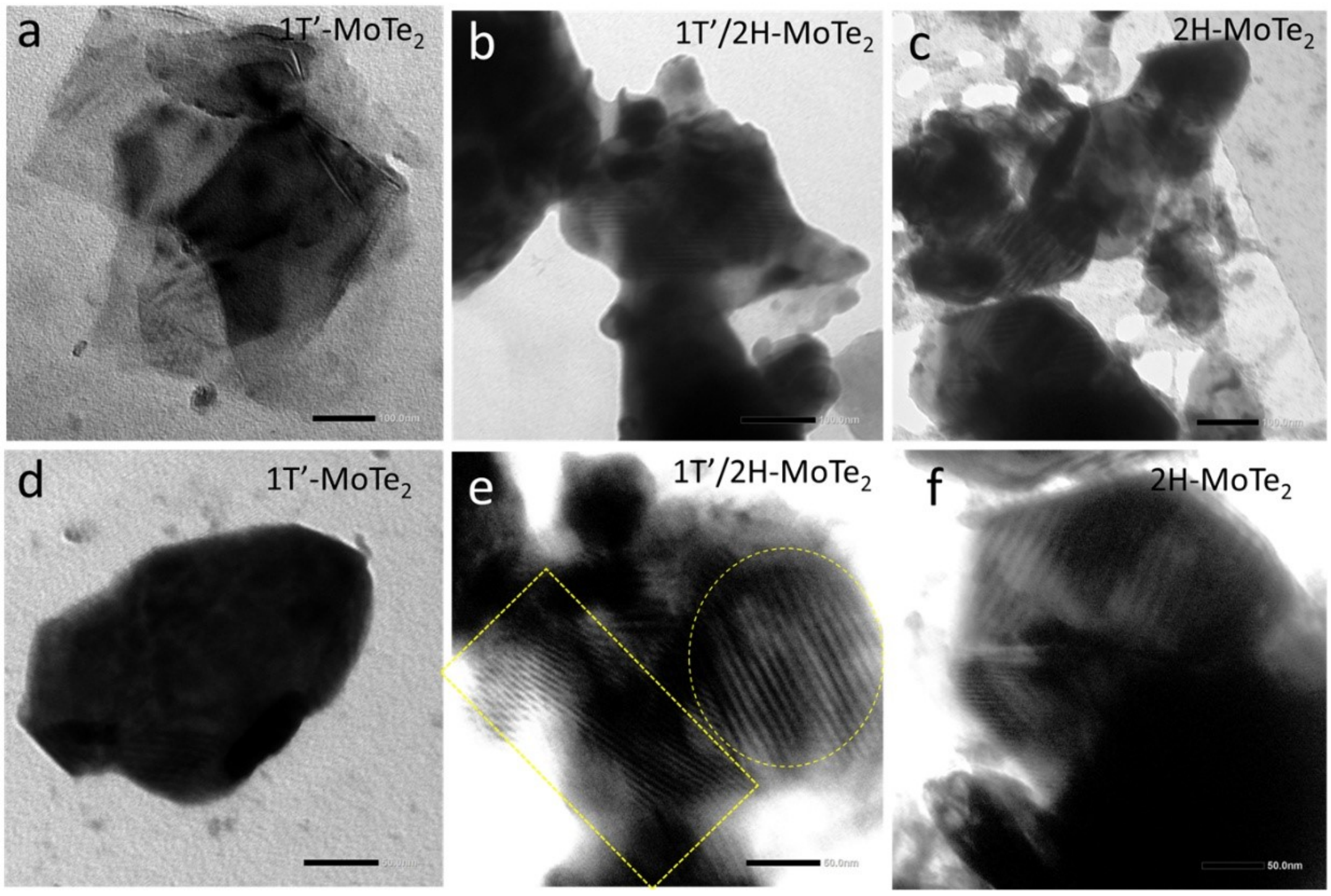

Figure S2. TEM images of (a, d) 1T'-$MoTe_2$, (b, e) mixed-phase 1T'/2H-$MoTe_2$ and (c, f) 2H-$MoTe_2$.

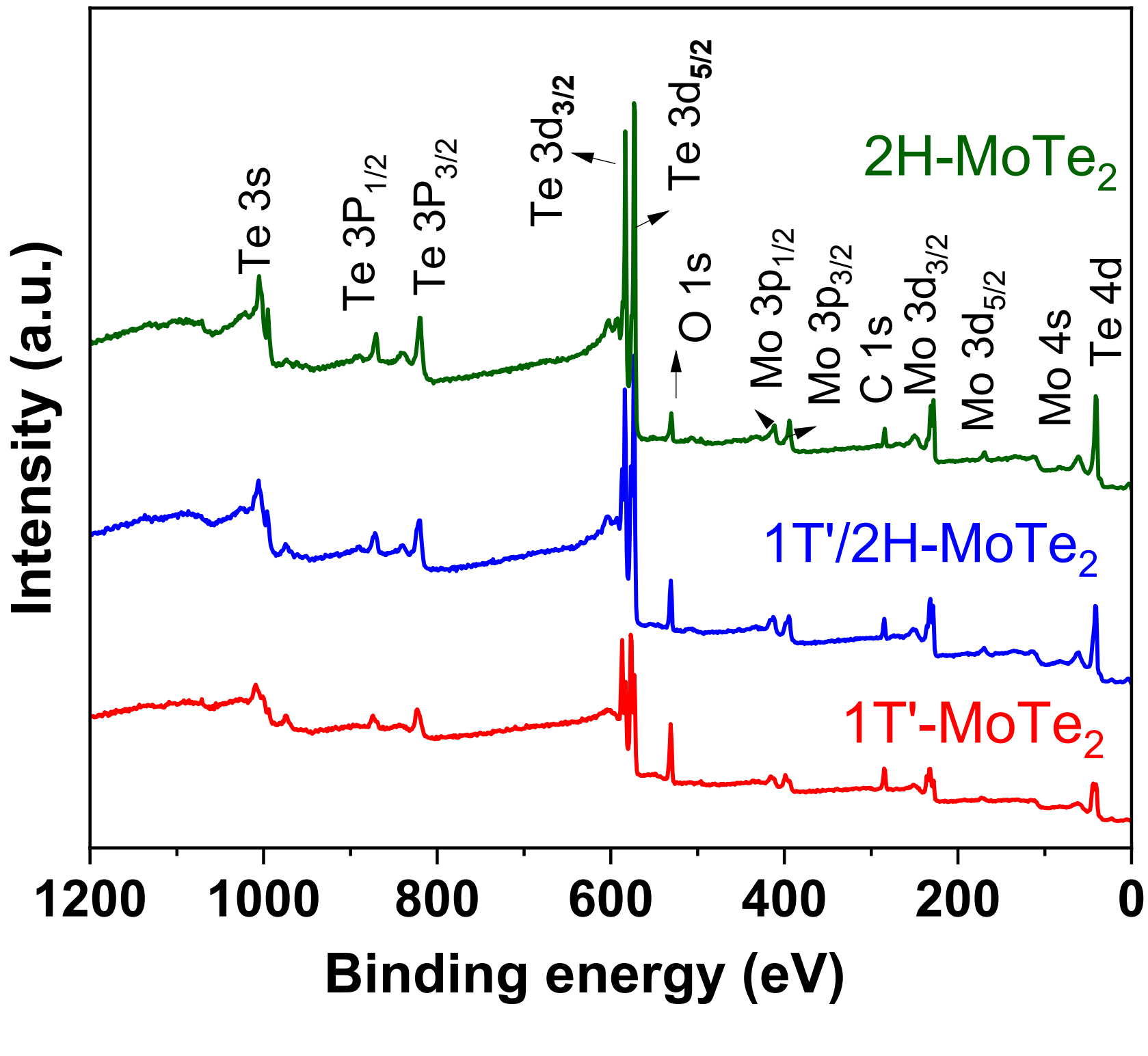

Figure S3. Wide-scan XPS spectra of 1T'-$MoTe_2$, 1T'/2H-$MoTe_2$, and 2H-$MoTe_2$.

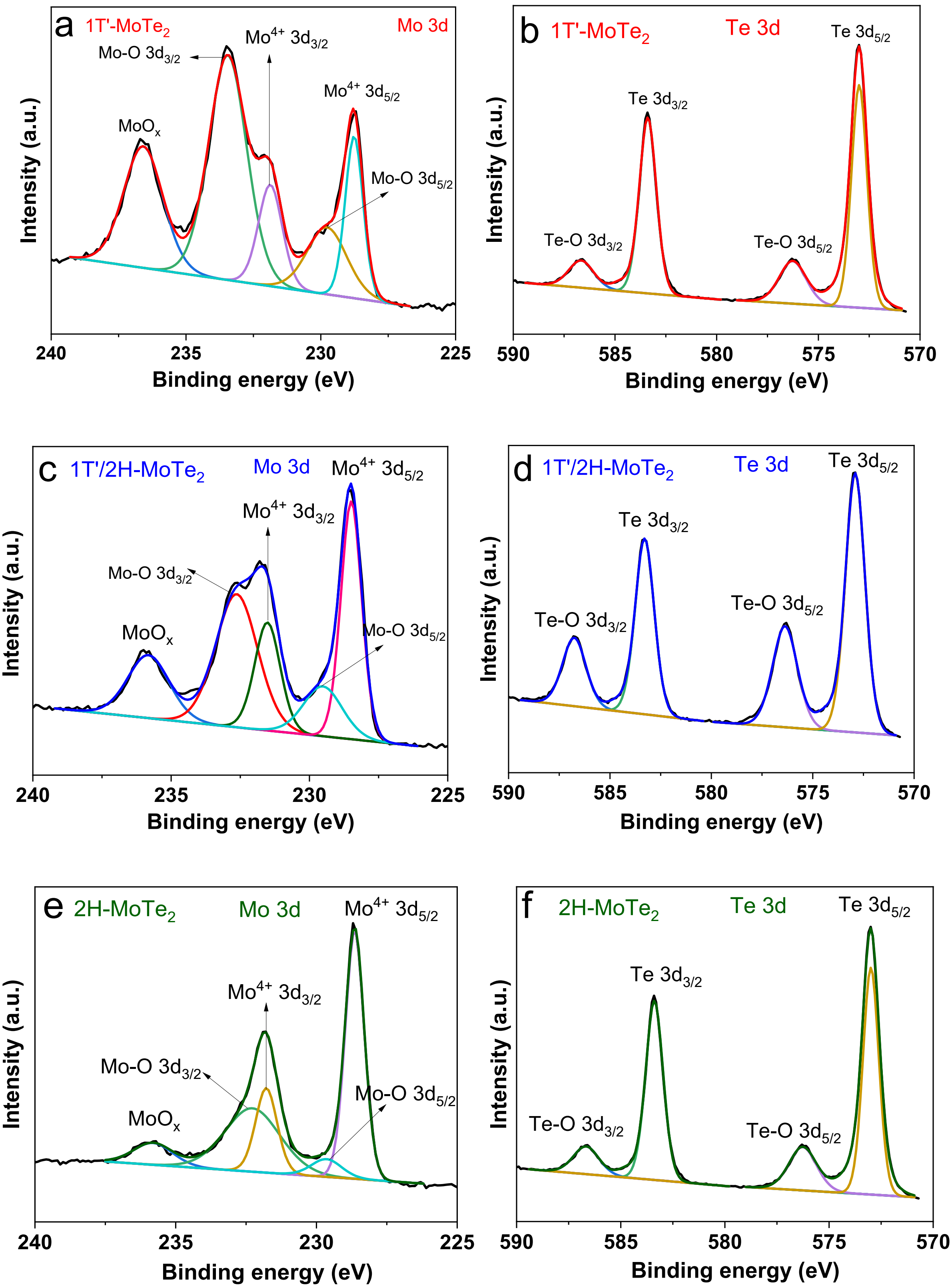

Figure S4. High resolution XPS spectra of Mo 3d and Te 3d peaks for **(a, b)** 1T'-$MoTe_2$, **(c, d)** 1T'/2H-$MoTe_2$, and **(e, f)** 2H-$MoTe_2$ catalysts.

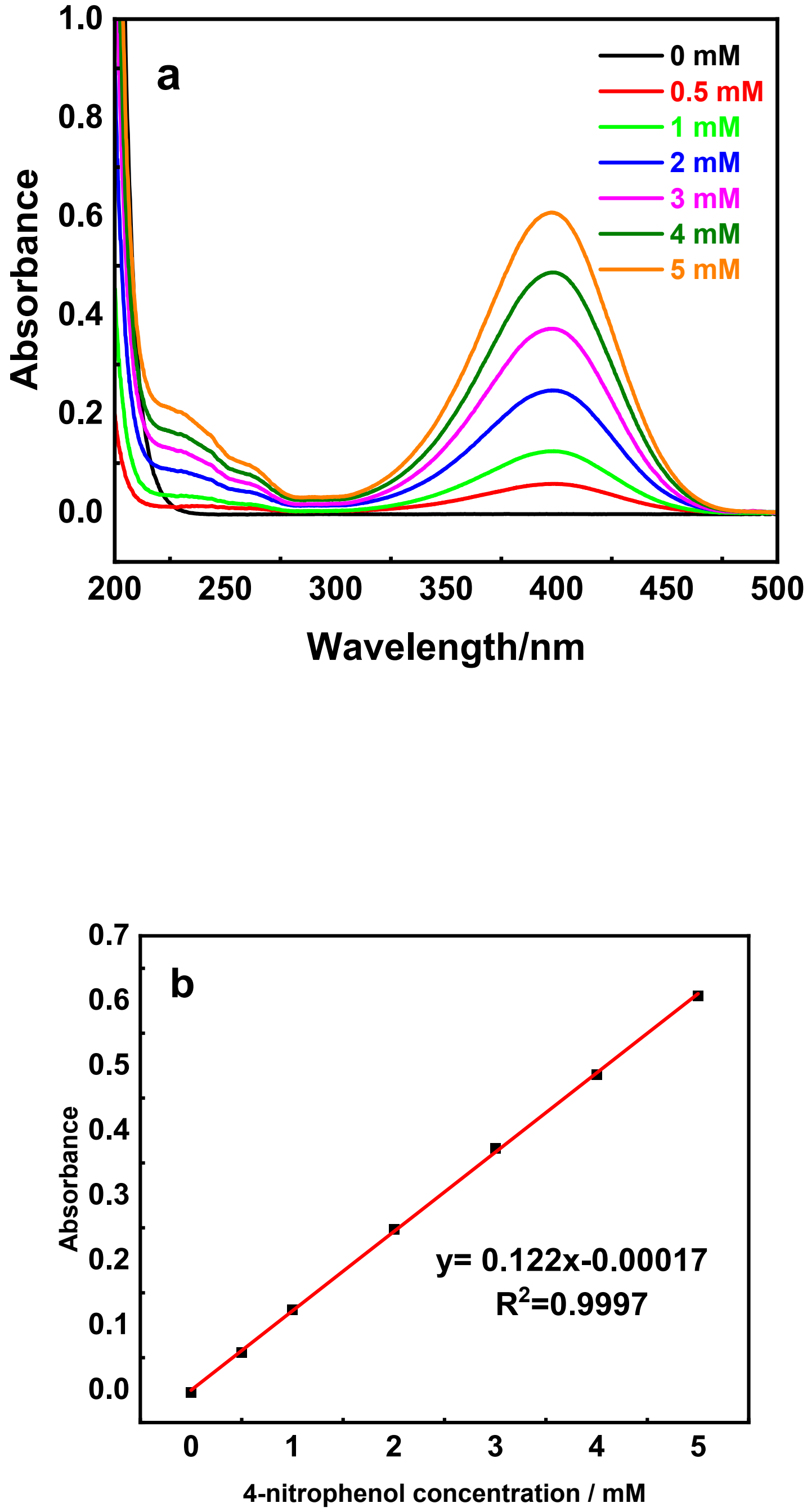

Figure S5. UV-visible absorption spectra (a) and calibration curve (b) of 4-nitrophenol with different concentrations.

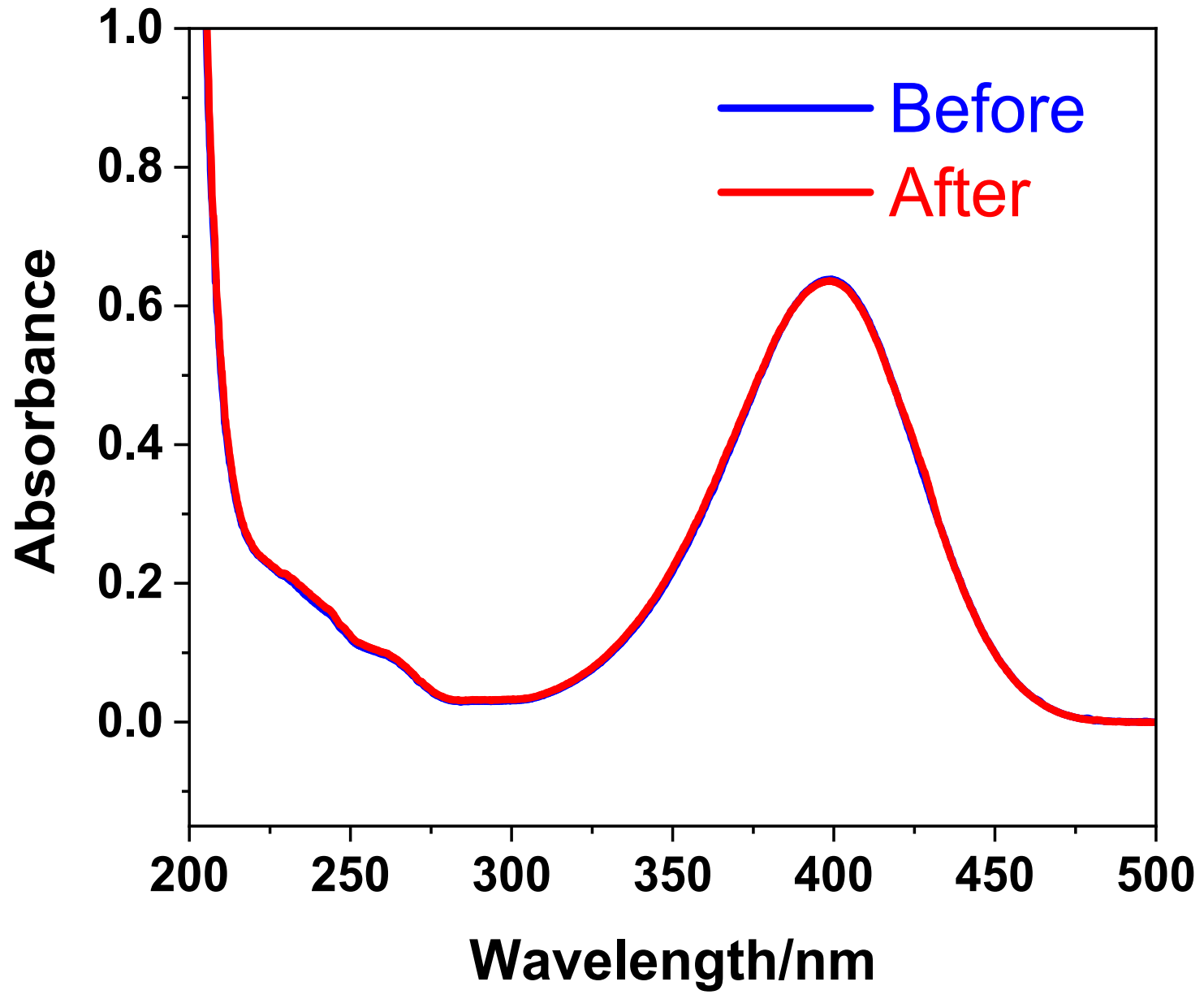

Figure S6. UV–visible absorption spectra of the control solution (10 mL of 0.5 mM 4-nitrophenol and 50 mg $NaBH_4$) before and after 15 minutes of stirring.

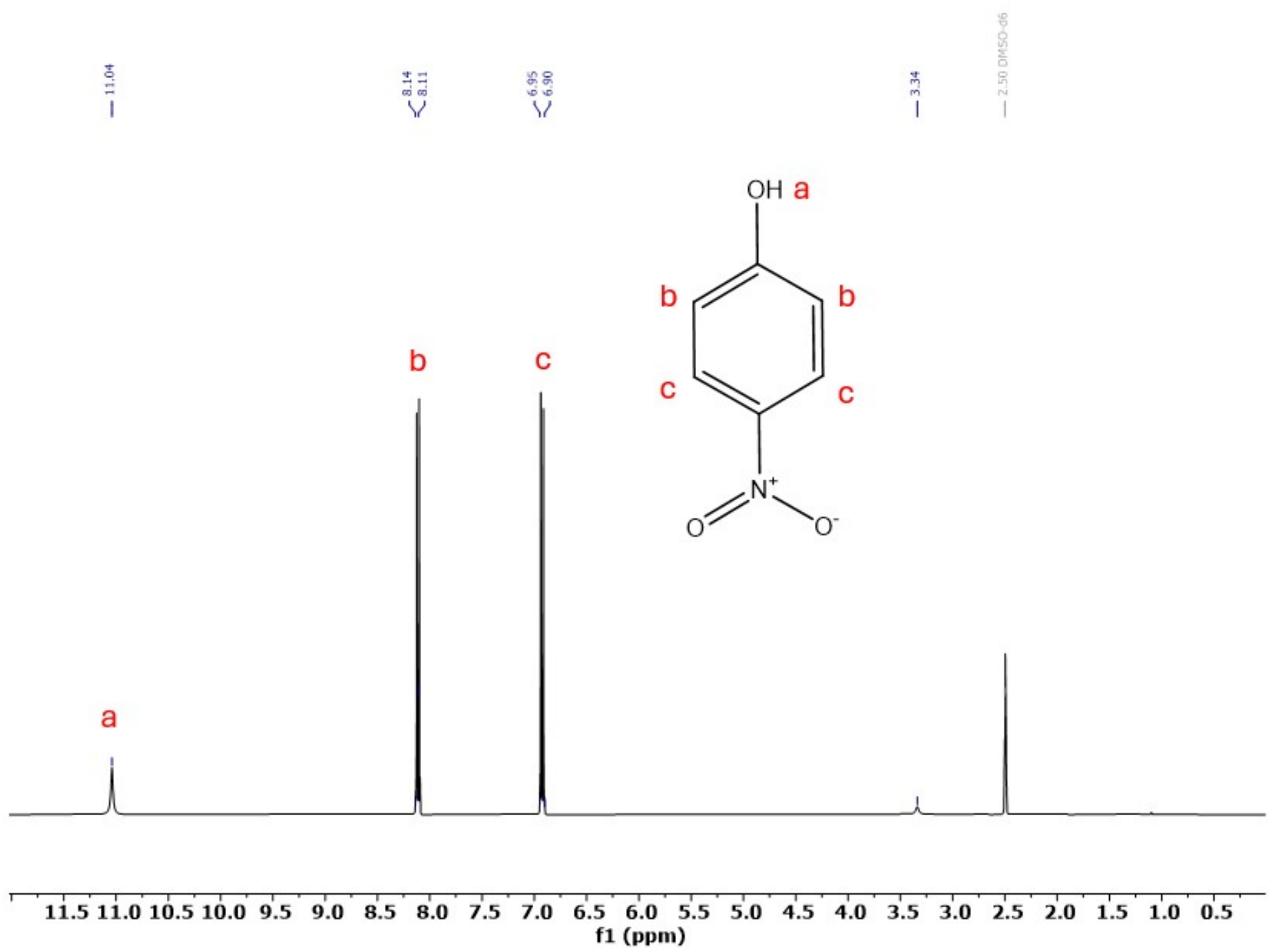

Figure S7. ^{1}H-NMR spectrum of 4-nitro phenol in DMSO-d6.

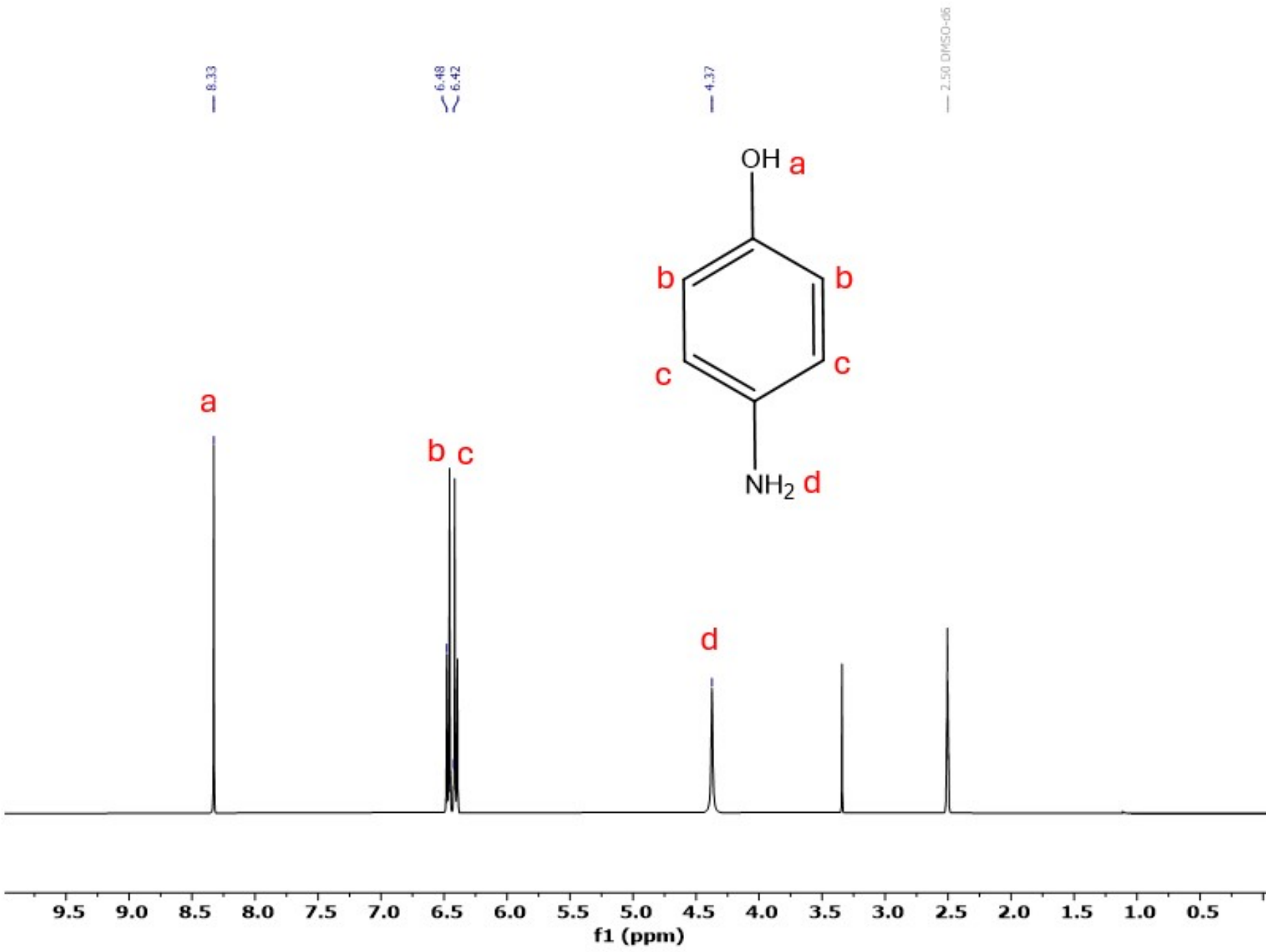

Figure S8. ^{1}H-NMR spectrum of 4-amino phenol in DMSO-d6.

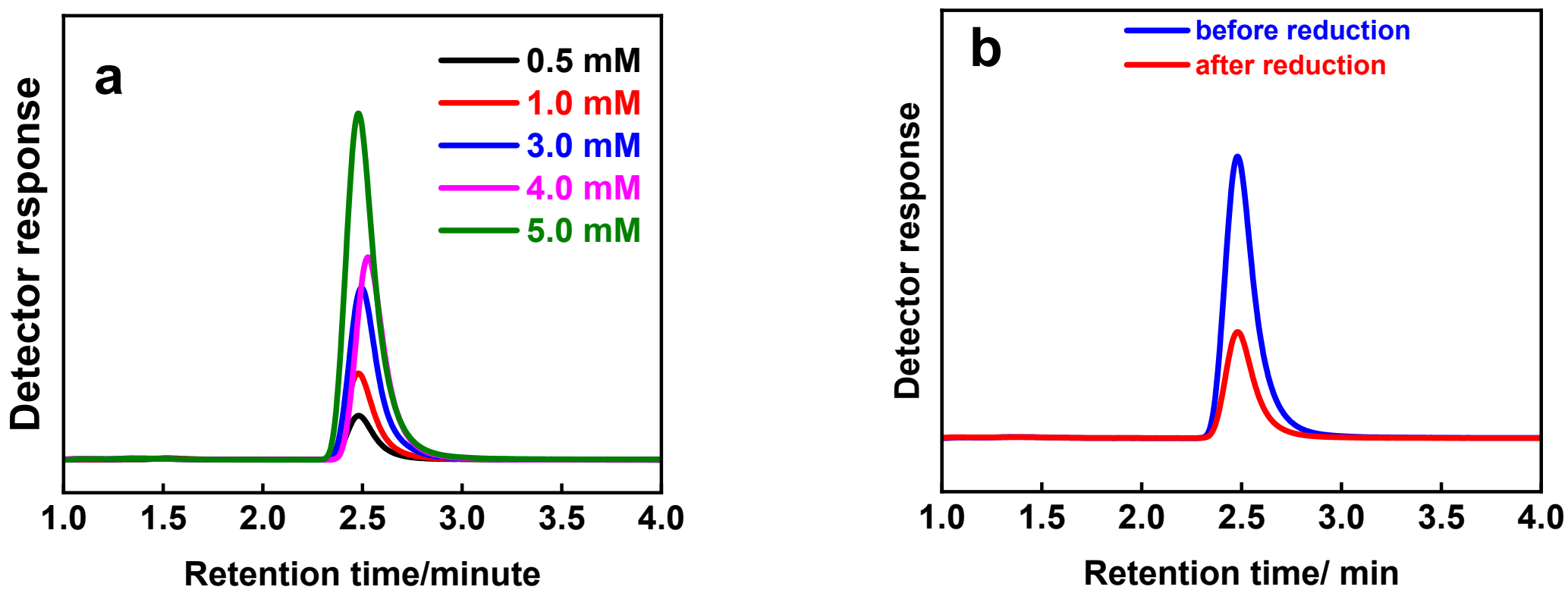

Figure S9. LC-MS chromatograms of stock solutions of 4-nitrophenol (0.5,1.0, 3.0, 4.0 and 5.0 mmol) with absorbance at $\lambda = 278$ nm are plotted vs. retention time **(a)**, and LC-MS spectra of the solution before (10 mL of 0.5 mM 4-nitro phenol and 0.13 mM $NaBH_4$) and after adding 1.42μM 1T'/2H-$MoTe_2$ catalyst followed by stirring for 15 minutes, and filtration **(b)**.

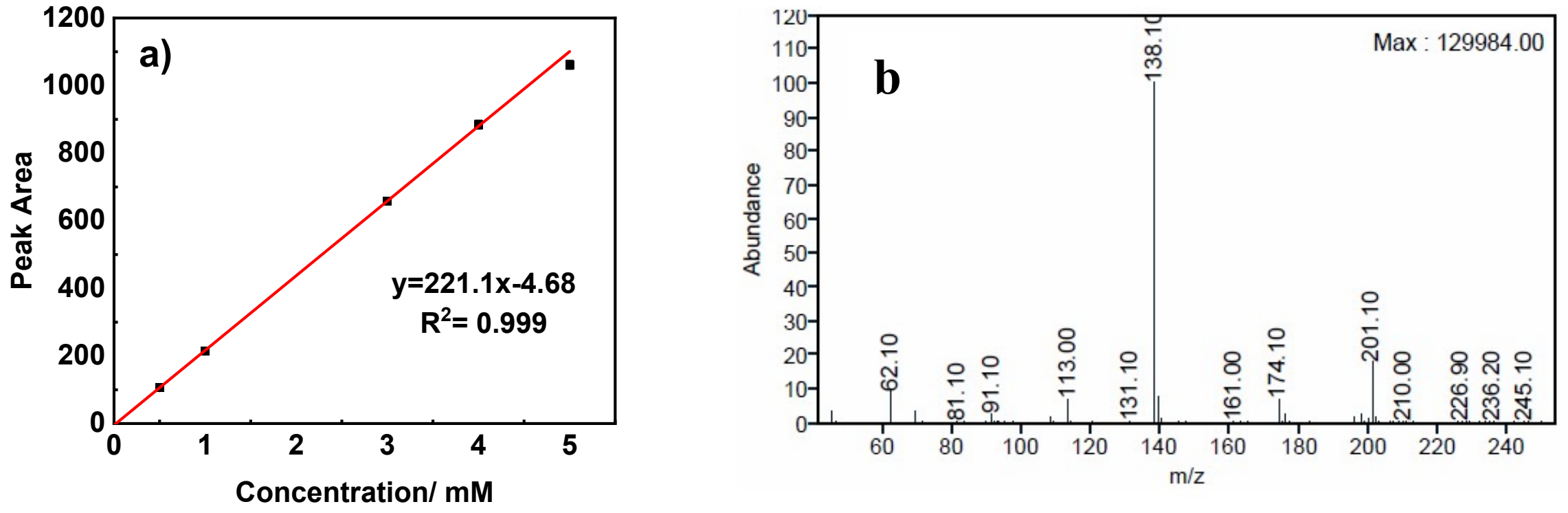

Figure S10. LC-MS chromatograms of stock solutions of 4-nitrophenol (0.5. 1.0., 3.0, 4.0 and 5.0 mmol) with absorbance at λ = 278 nm, with peak area (at retention time = 2.64 min) plotted vs. the concentration of 4-nitro phenol **(a)**, and the corresponding mass spectrum for the characteristic peak of the chromatogram, showing a signal for ($[M-H]^-$, m/z = 138) **(b)**.

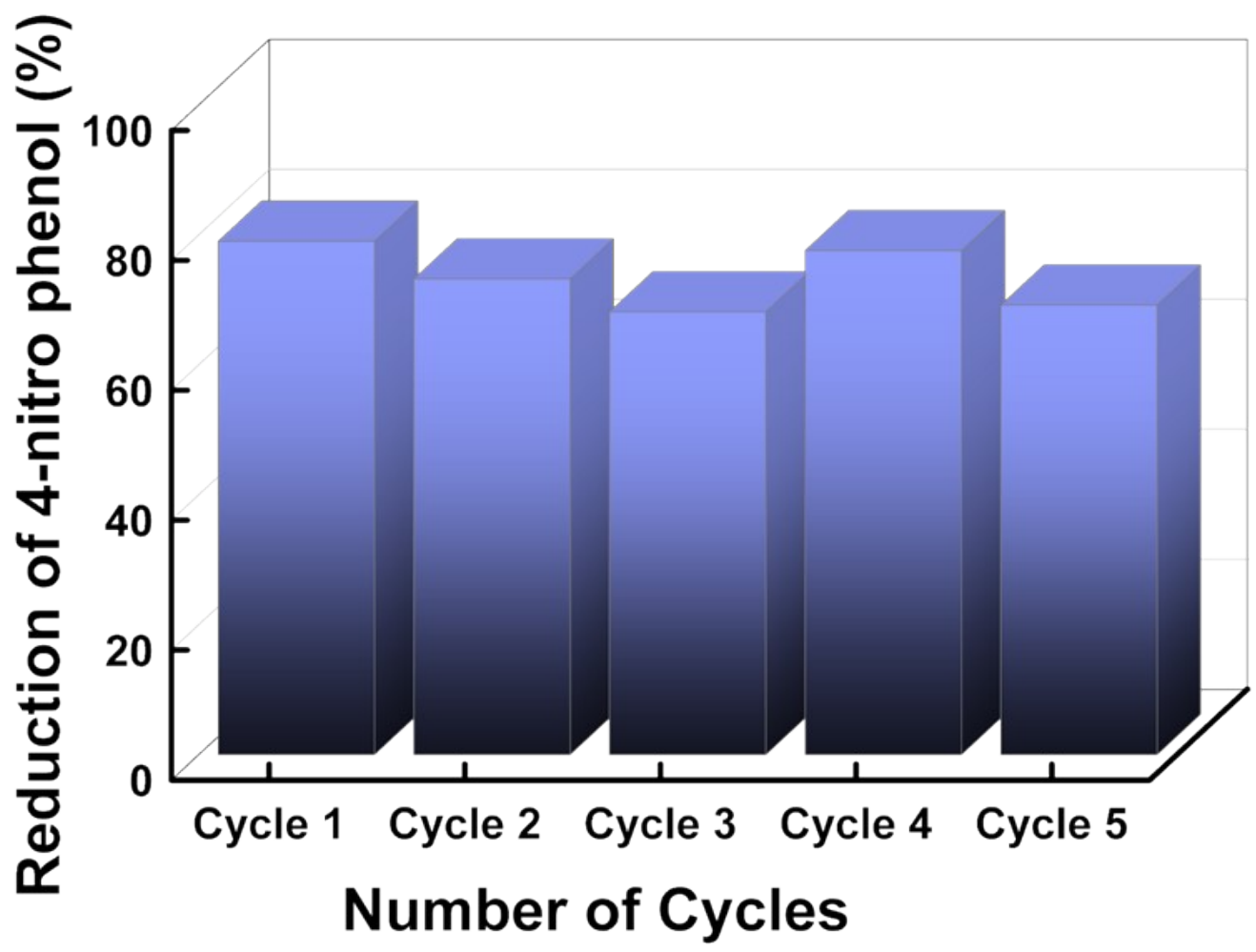

Figure S11. The reduction of 4-nitro phenol using mixed-phase 1T'/2H-$MoTe_2$ over 5 cycles of reuse.

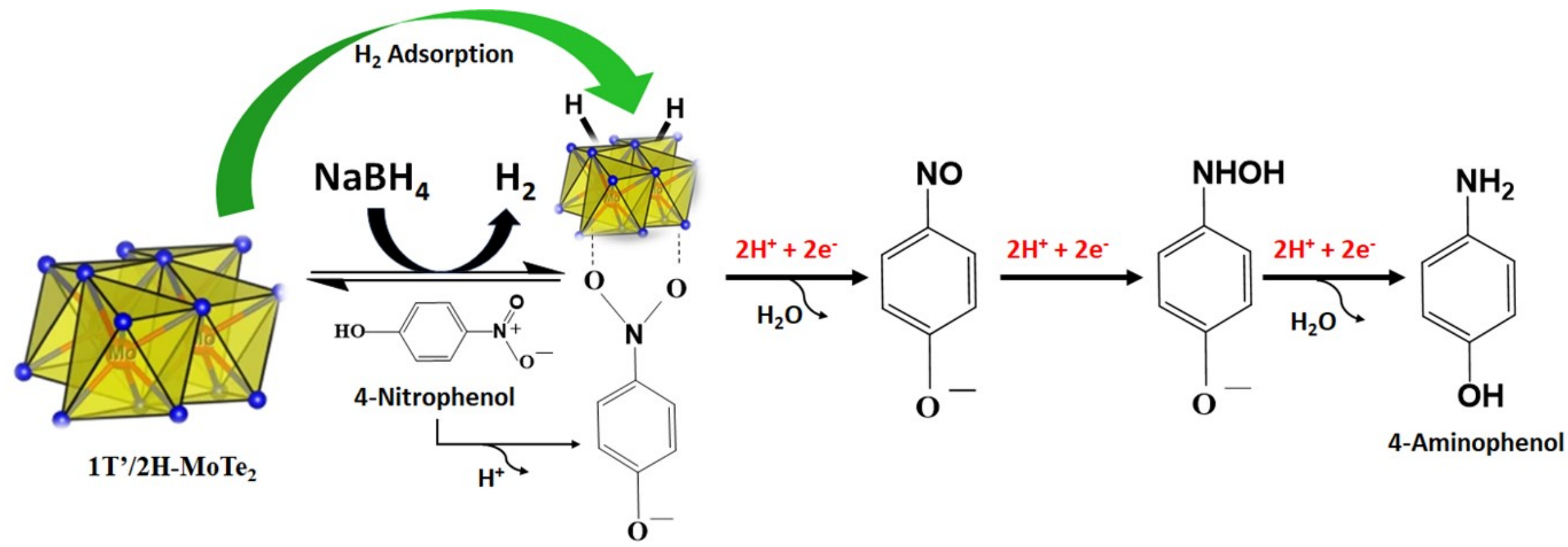

Figure S12. Mechanism for the reduction of 4-nitrophenol to 4-aminophenol using the mixed 1T'/2H-$MoTe_2$ catalyst.

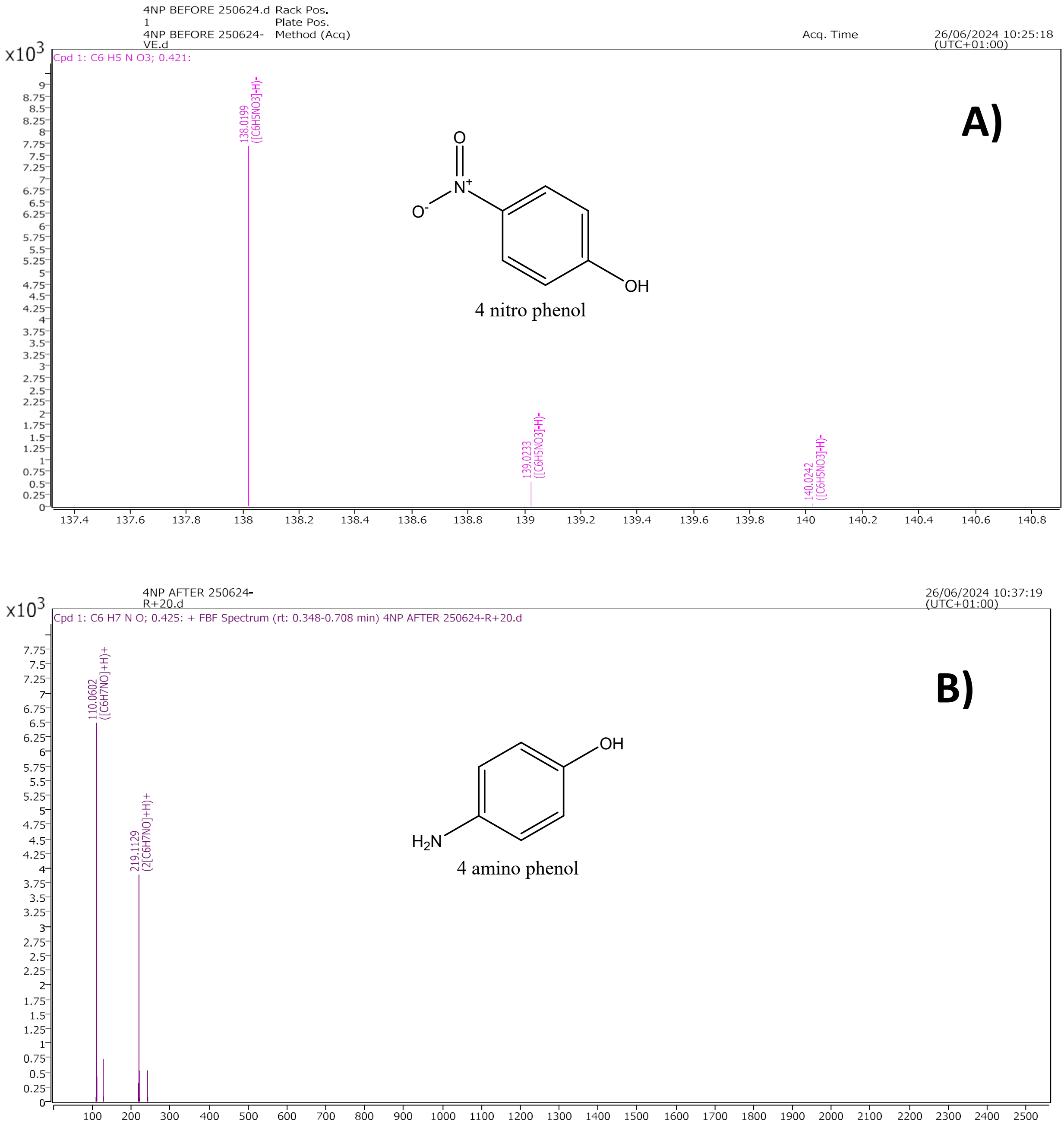

Figure S13. (a) Mass spectrum of 4-nitro phenol before reaction with selected portions of the spectrum enlarged to show the molecular ion (m/z = 138), and (b) after the reduction of 4-nitro phenol using mixed 1T'/2H-$MoTe_2$ for 15 minutes, with selected portions of the spectrum enlarged to show the molecular ion (m/z = 110).

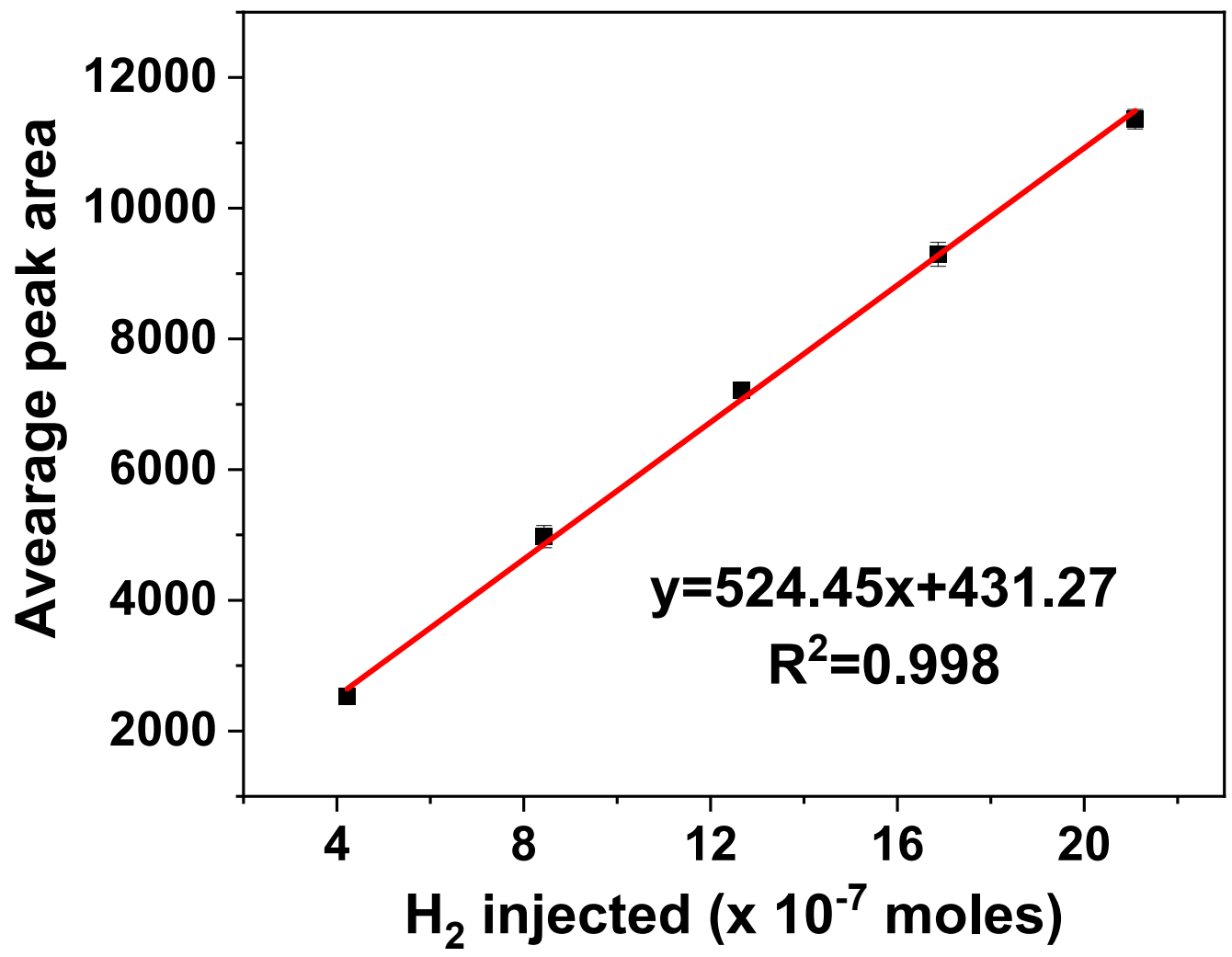

Figure S14. Gas chromatography calibration curve for H_2.

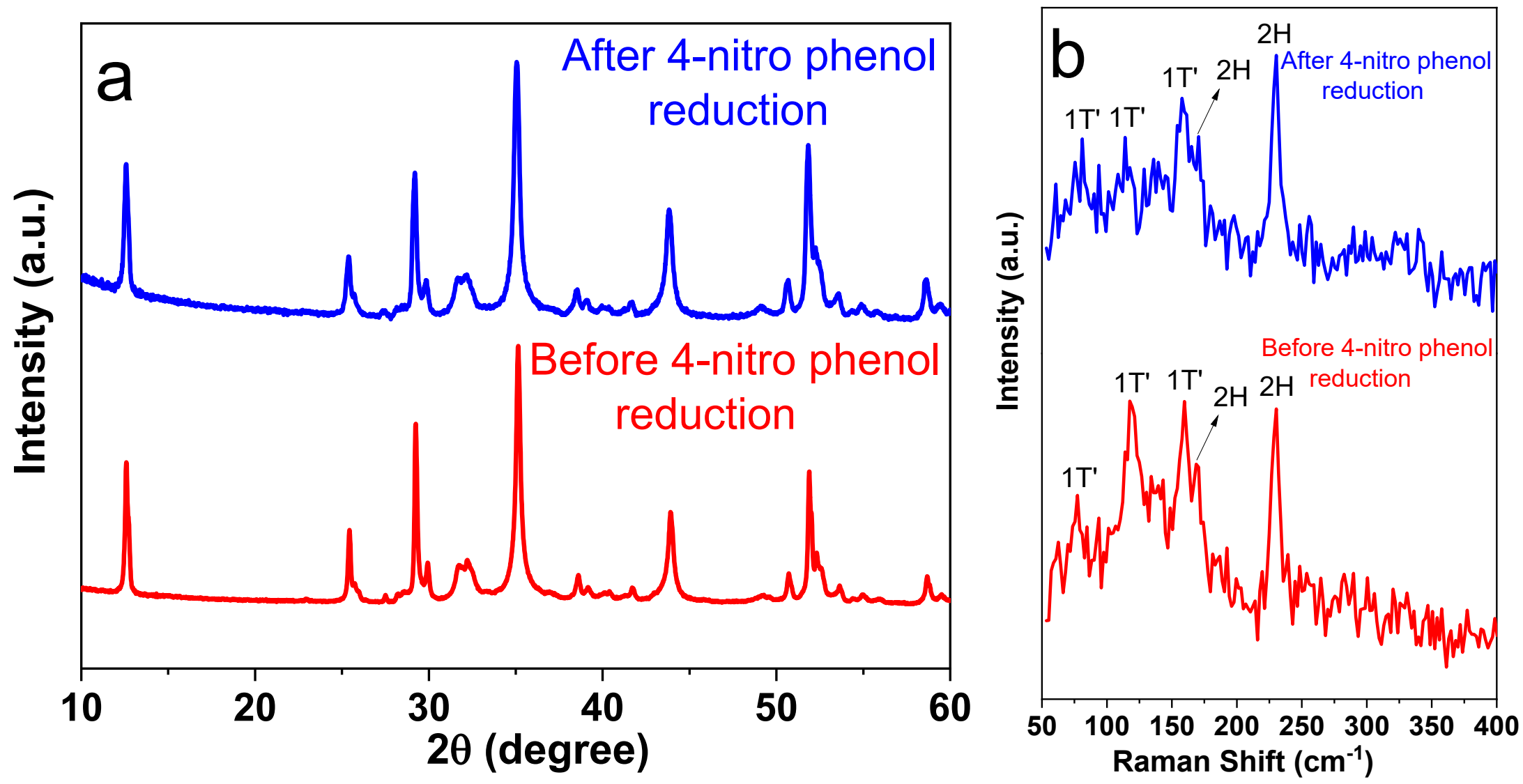

Figure S15. Post experiemntal **(a)** XRD and **(b)** Raman analyses shows the structural stability of the 1T'/2H-$MoTe_2$ catalyst before and after 5 cycles of 4-nitro phenol reduction.

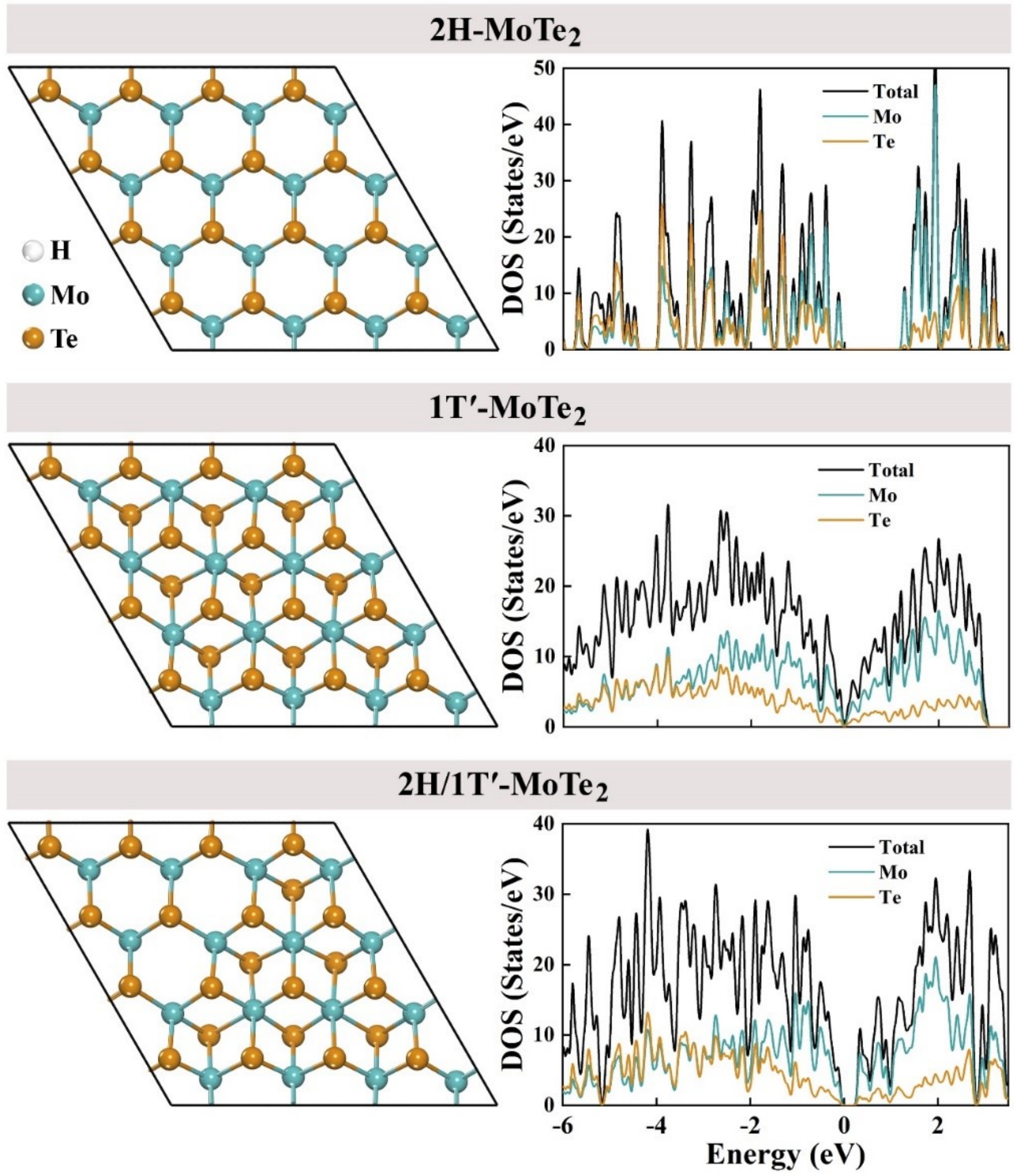

Figure S16. Optimized structures and corresponding partial density of states using the HSE functional for $MoTe_2$ polymorphs with 2H, 1T', and 2H/1T' phases.
